# Quantifying Spatial variation in environmental and sociodemographic drivers of leptospirosis in the Dominican Republic using a geographically weighted regression model

**DOI:** 10.1038/s41598-025-13413-5

**Published:** 2025-07-25

**Authors:** Beatris Mario Martin, Benn Sartorius, Helen J. Mayfield, Angela M. Cadavid Restrepo, Behzad Kiani, Cecilia Jocelyn Then Paulino, Marie Caroline Etienne, Ronald Skewes-Ramm, Michael de St Aubin, Devan Dumas, Salome Garnier, William Duke, Farah Peña, Gabriela Abdalla, Lucia de la Cruz, Bernarda Henríquez, Margaret Baldwin, Adam Kucharski, Eric J. Nilles, Colleen L. Lau

**Affiliations:** 1 Faculty of Health, Medicine, and Behavioural Sciences, University of Queensland Centre for Clinical Research (UQCCR), The University of Queensland, Brisbane, QLD 4029 Australia; 2 School of Public Health, Faculty of Health, Medicine, and Behavioural Sciences, The University of Queensland, Brisbane, QLD 4029 Australia; 3 Ministry of Health and Social Assistance, Santo Domingo, Dominican Republic; 4 Brigham and Women’s Hospital, Boston, MA USA; 5 Harvard Humanitarian Initiative, Cambridge, MA USA; 6 Pedro Henríquez Ureña National University, Santo Domingo, Dominican Republic; 7 London School of Hygiene & Tropical Medicine, London, UK; 8 Harvard Medical School, Boston, MA USA

**Keywords:** Zoonosis, Seroprevalence survey, Risk factors, Spatial regression, Caribbean region, Infectious diseases, Computational models

## Abstract

Spatial variation in drivers of leptospirosis transmission in the Dominican Republic is poorly understood. To inform targeted public health interventions, we aimed to characterise risk factors and drivers of transmission on a fine spatial scale. We analysed data from 2078 participants in two provinces, Espaillat and San Pedro de Macoris (SPM), collected from a 2021 cross-sectional survey. We used geographically weighted regression to quantify associations between leptospirosis seropositivity and spatial environmental and sociodemographic data. In Espaillat, higher odds of seropositivity were associated with exposure to freshwater (OR 6.51, ranging between 5.95 and 6.99), bare ground coverage above 1.44% (OR 3.70;3.49–3.92), river density surrounding the household at the highest quartile (OR 2.43;2.41–2.45) and average precipitation in the last five years at the highest quartile (2.57; 2.52–2.65). In SPM, rat exposure was associated with higher odds of seropositivity (OR 2.59;1.23–3.14). By identifying locally important drivers of transmission, our study provides evidence to support more tailored public health interventions to optimise the control and prevention of leptospirosis.

**Supplementary Information:**

The online version contains supplementary material available at 10.1038/s41598-025-13413-5.

## Introduction

Leptospirosis is a globally distributed zoonotic disease caused by pathogenic species of the *Leptospira* bacteria^1^. An estimated 1.03 million human leptospirosis cases and 58,900 deaths occur annually around the world^2^. Latin America and the Caribbean account for one-third of all globally reported leptospirosis outbreaks^3^. Estimated annual morbidity varies considerably, ranging from 3.9/100,000 population in South America to 50.7/100,000 population in the Caribbean^2^. Human infection is primarily acquired through direct contact with urine or tissues of infected animals, or indirect exposure to contaminated soil or water^1^. In tropical regions, the combination of warm climates, high rainfall and humidity, and informal settlements with limited infrastructure and sanitation access provides favourable conditions for leptospirosis transmission. The two primary epidemiological profiles include urban outbreaks triggered by heavy rainfall, flooding and other natural disasters that predominantly affect areas with poor infrastructure; and rural outbreaks primarily linked to endemic occupational exposures common in resource-poor areas^4^.

In 2020, the Dominican Republic (DR) reported 210 leptospirosis cases and 38 related deaths^5^. Accurate population-level prevalence data are critical for effective public health planning and interventions. Moreover, locally based interventions should take into account that the occurrence of leptospirosis reflects the complex interaction among humans, reservoir animals and the environment^6^, which varies across space. Statistical models that explore transmission risk factors and drivers of leptospirosis need to account for this spatial heterogeneity. Spatial models can therefore provide a more comprehensive understanding of disease patterns, allowing more informed and efficient decision-making. An enhanced population-level understanding of leptospirosis distribution can ensure that high-risk populations and locations are prioritised for support, optimizing the use of limited resources and potentially reducing overall health costs and health disparities.

Results from our previous study in two target provinces showed an adjusted leptospirosis seroprevalence of 11.3% (95%CI10.8-13.0), using the microscopic agglutination test (MAT)^7^. In the current study, we expand on this prior work by incorporating spatial data into our analysis and using spatially explicit statistical models. This study aimed to investigate spatial variation in environmental and sociodemographic risk factors and drivers of leptospirosis seroprevalence at the household level in the DR using generalised geographically weighted regression (GGWR) modelling. Our objective was to characterise risk factors and drivers of transmission on a fine spatial scale, which can be leveraged to inform tailored local public health interventions.

## Methods

### Setting

The DR is in the Caribbean and occupies two-thirds of the island of Hispaniola, which it shares with Haiti. Due to its location and geophysical characteristics, the DR experiences frequent extreme weather events, including hurricanes and tropical storms^8^. In local vulnerable areas, flooding, landslides and other natural disasters can lead to negative socioeconomic consequences and significant disease outbreaks^9^.

The DR is the second most populous country in the Caribbean region, with an estimated population of ~ 10.5 million in 2020^5^. Nearly 80% of the population resides in urban or semi-urban areas, but only about 20% of communities are classified as urban^9^. In 2020, the population median age was 26.8 years with a ~ 50:50 male-to-female ratio^10^. The country is divided into 31 provinces plus the Santo Domingo National District and subdivided into 155 municipalities, 386 district municipalities, 1565 sections and 12,565 communities.

### Survey design

**Fig. 1:**
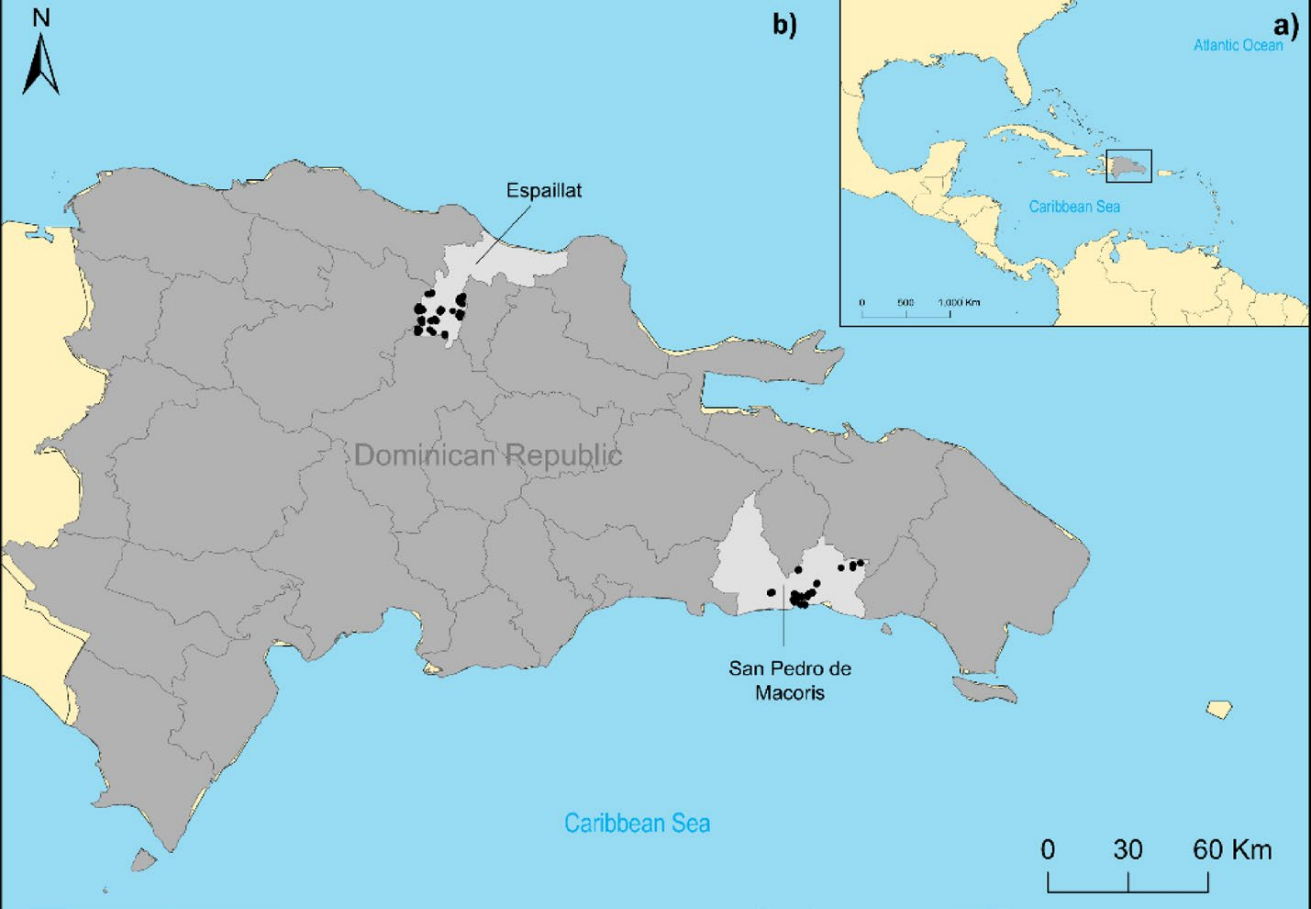
Map of the Caribbean region (**a**) and the Dominican Republic (**b**). In panel B the 31 provinces and the Municipal District Santo Domingo are shown, with the two provinces included in this study highlighted. Black dots represent the location of households included in the study. Maps were created in Esri^®^ ArcGIS software v 10.8 (https://www.esri.com/en-us/arcgis/products/arcgis-desktop/resources, Esri^®^ ArcMap 10.8.0.12790. Redlands, CA, USA)^13^, base layer from layer from United Nations OCHA – COD-AB dataset (https://data.humdata.org/dataset/cod-ab-dom), licensed under CC BY-IGO.

Between 30 June and 12 October 2021, a nationally representative cross-sectional serosurvey was conducted in the DR. A detailed description of the survey design and data collection has been previously reported^11^, and a summary of the study design has been included in the Supplementary information (Methods). The national survey included 6,683 participants, aged 6 to 97 years (median 40, interquartile range (IQR) 23–58 years), from all 31 provinces and the National District. In this study, we analysed data from two provinces, Espaillat in the northwest and San Pedro de Macoris (SPM) in the southeast. These two provinces were oversampled (*n* = 2091) in the national study as they were linked to an ongoing clinical surveillance study investigating acute febrile illnesses and therefore provide more spatially granular data suitable for the current study^12^ (Fig. 1).

### Survey data collection

A trained field team interviewed and collected venous blood from all participants and captured Global Positioning System (GPS) coordinates of each household. Survey data collection procedure has been previously described^11^ and full details on how variables were defined and measured are available in the Supplementary information (Methods). The interviews were conducted in Spanish, and Creole questionnaires and Creole speakers were available if requested. A questionnaire was used to collect data from each individual (including demographics, occupation, education, etc.). For each household, a separate questionnaire was used to collect household-level data (access to piped water, materials used on the floor, vehicle ownership, etc.) from one household representative, and their answers were linked to all members of that household.

Venous blood samples were processed as sera and frozen at −80 °C. MAT was used to detect anti-*Leptospira* antibodies. Serological analyses were performed at the US Centers for Disease Control and Prevention’s Zoonoses and Select Agent Laboratory, Bacterial Special Pathogens Branch, Atlanta, GA, USA. A panel of 20 pathogenic serovars were selected for the MAT panel, and titres of ≥ 1:100 were considered seropositive and indicative of prior infection.

### Spatial data collection

Based on conceptual leptospirosis transmission frameworks^6^, we integrated environmental, sociodemographic and census data with our survey data. Land cover was aggregated into five groups; *crops* correspond to human-planted cereals, grasses and crops; *rangeland* to open areas covered with homogeneous grasses; *bare ground* to areas of rock or soil with very sparse to no vegetation; *trees* to areas of dense vegetation; and *built-up* to human-made structures, major roads and rail networks (Supplementary Table 1). Characteristics (e.g., resolution, distribution) of the spatial data considered for the analyses, data extraction and aggregation are described in the Supplementary information (Supplementary Figs. 1–5, Supplementary Table 2). Using Esri^®^ ArcGIS software v 10.8 (Esri^®^ ArcMap 10.8.0.12790. Redlands, CA, USA)^13^, spatial data layers (vector or raster formats) were overlaid with the survey data for data extraction. The survey data were represented as a vector layer, with each point-data signifying the geographical location of a surveyed household.

### Data analysis

Generalised mixed-effect regression (GLMER) and GGWR were used to assess and quantify the odds ratio (OR) for leptospirosis seropositivity associated with each covariate. We employed a two-stage variable selection process to identify variables for inclusion in the final GLMER and GGWR models, conducted separately for each province. The variables retained in the final models were selected based on biological plausibility. In the first stage, bivariate mixed-effect models were fitted for each province separately (and combined) with the sampling community level included as a random effect. During the variable selection process, we identified that differences in seroprevalence between the two provinces were impacting the model (i.e. the results of the model with data from the two provinces combined, suggested all variables that occurred more frequently in the province with higher prevalence to be positively associated with leptospirosis seropositivity), thus we generated separate models for each region. Additionally, given the considerable geographic distance between the two provinces, applying one model to a combined dataset could not be appropriate. Variables with a *P*-value below 0.20 were included in the preliminary multivariable GLMER (Supplementary Table 3). Collinearity was assessed using the Variance Inflation Factor (VIF), and any variables with a VIF exceeding 10 in any multivariable model were excluded (Supplementary Table 4, Supplementary Tables 5, and Supplementary Table 6). Starting with the variables with the highest VIF, each was individually excluded until the VIF of all remaining variables was less than 10. As the variables included in each province model were selected independently, the final set of variables was province-specific (Supplementary Table 7). All variables selected for inclusion in the final models were investigated for a nonlinear relationship with leptospirosis seropositivity using a generalised additive model (GAM). Variables with nonlinear association were categorised in quartiles if they were homogeneously distributed across participants. Variables that were heterogeneously distributed were inspected using density distribution histograms to identify the optimal categorisation (Supplementary Methods).

We used a Bayesian hierarchical shrinkage to build the GLMER models; associations were considered statistically significant if the 95% confidence interval (95%CI) of the estimated OR excluded one. For the GGWR models, results are shown as the median, minimum and maximum OR. R statistical programming language (R version 4.1.3, 2022-03-10)^14^ was used for mixed effects models (brms) and GGWR models (*GWmodel*) (Supplementary Table 8).

### Ethics approval and consent to participate

The 2021 field survey ethical approval was obtained from the National Council of Bioethics in Health (013-2019), the Institutional Review Board of Pedro Henríquez Ureña National University, Santo Domingo, DR; the Mass General Brigham Human Research Committee, Boston, USA (2019P000094); and the Human Research Ethics Committee of The University of Queensland (2022/HE001475), Brisbane, Australia. This research was conducted in accordance with the Declaration of Helsinki.

#### Informed consent

was obtained from all participants. For participants < 18 years old, except emancipated minors, consent was obtained from the parent or legal guardian. Participants between 14 and 17 years old provided written assent and those between 7 and 13 years old provided verbal assent. For participants between 6 and 7, only parental consent was obtained. Study procedures and reporting adhered to the STROBE criteria for observational studies.

## Results

After excluding participants with missing data, a total of 2,078 study participants from 23 communities across the two provinces were included in this analysis. The median age was 39 (23,56) years, 1,329 (64.0%) were female, and 43.5% were from rural communities. Overall, 237 (11.4%) participants were seropositive. Characteristics of participants from each province are shown in Table 1. 50% of participants included in SPM were under 35 years old, while in Espaillat the number of participants across the age groups was more evenly distributed. In Espaillat, a higher proportion of participants self-reported being farmers (7.4%) compared to SPM (1.2%), although the proportion who worked in outdoor environments was similar in both provinces. In Espaillat Province, 127 (15.8%) participants were seropositive and in SPM Province 110 (8.6%).

**Table 1:** Characteristics of the study population by province, Dominican republic, 2021.

Population characteristics	Overall^1^	Espaillat	San Pedro de Macoris
**Overall**	2078 (%)	802 (%)	1 276 (%)
**Age (years)**			
**Median (IQR)**	39 (23, 56)	44 (28, 61)	34 (21, 52)
**Category**			
5–19	386 (18.6)	101 (13)	285 (22)
20–34	530 (25.5)	176 (22)	354 (28)
35–49	459 (22.1)	191 (24)	268 (21)
50–64	392 (18.9)	174 (22)	218 (17)
65+	311 (15.0)	160 (20)	151 (12)
**Gender**			
Female	1329 (64.0)	501 (62)	828 (65)
Male	733 (35.3)	297 (37)	436 (34)
Other	16 (< 1)	4 (< 1)	12 (< 1)
**Occupation**			
Farmer	﻿74 (3.6)	59 (7·4)	15 (1·2)
Not-farmer	2004 (96.4)﻿	743 (93)	1,261 (99)
**Work Environment**			
Indoor or Student or houseperson	1148 (55.2)	447 (56)	701 (55)
Outdoor	92 (4.4)	34 (4·2)	58 (4·5)
Mixed indoor and outdoor	289 (13.9)	158 (20)	131 (10)
Retired or unemployed	549 (26.4)	163 (20)	386 (30)
**Maximum educational level**			
Primary or none	658 (31.7)	272 (34)	386 (30)
Secondary, tertiary or technical	1420 (68.3)	530 (66)	890 (70)
**Ethnicity**			
Indigenous	326 (15.7)	99 (12)	227 (18)
Mestizo	667 (32.1)	263 (33)	404 (32)
Mulatto	1027 (49.4)	389 (49)	638 (50)
White or other	58 (2.8)	51 (6)	7 (< 1)
**Setting**			
Rural	904 (43.5)	467 (58)	437 (34)
Urban	1174 (56.5)	335 (42)	839 (66)
**Rat exposure**			
No	1747 (84.1)	798 (100)	949 (74)
Yes	331 (15.9)	4 (< 1)	327 (26)

### Non-spatial and spatial models

In each province, the GLMER identified a different set of significant environmental and sociodemographic variables associated with leptospirosis seropositivity (Tables 2 and 3). Within and between each province, the GGWR models identified substantial spatial variation in the OR of leptospirosis seropositivity associated with each covariate. The range of variation and direction of association change between provinces and detailed results are presented below.

### Espaillat

In the multivariable GLMER, variables associated with significantly higher OR of leptospirosis seropositivity included older age groups (reference 5–19 years), namely: 20–34 OR 3.66 (95%CI 1.07–13.72); 35–49 years OR 4.48 (1.24–17.91); 50–64 years (OR 3.95;1.07–16.24); and ≥ 65 years OR 11.804 (3.01-61.18). Male gender (OR 3.40;1.60–7.89), and exposure to freshwater (OR 13.33;1.20-166.6) also emerged as significant risk factors. The OR increased significantly with river density within a 250-meter buffer surrounding the household at the highest quartile (OR ﻿6.78; 1.94–29.51)  and average precipitation in the last five years at the highest quartile (OR ﻿5.41; 1.39–24.81)  (Table 2).

**Table 2:** Odds ratios (ORs) and 95% CI from the generalised linear mixed-effects regression (GLMER) and OR median and range from the generalised geographically weighted regression (GGWR) for leptospirosis seropositivity in Espaillat province, Dominican republic, 2021.

	GLMER OR (95%CI)	Median (Min·-Max·)
Age (years)		
15–19		
20–34	**3.66 (1.07–13.72)**	2.95 (2.86–3.04)
35–49	**4.48 (1.24–17.91)**	3.38 (3.15–3.57)
50–64	**3.95 (1.07–16.24)**	3.57 (3.19–3.84)
≥ 65	**11.80 (3.01–61.18)**	4.71 (4.30–5.01)
**Gender**		
Female		
Male	**3.40 (1.60–7.89)**	2.09 (2.01–2.18)
Other	3.47 (0.15–75.62)	5.55 (5.39–5.83)
**Ethnic group**		
Indigenous		
White or other	0.30 (0.04–1.91)	0.57 (0.53–0.65)
Mestizo	0.55 (0.18–1.79)	0.65 (0.63–0.67)
Mulatto	0.58 (0.18–1.89)	0.66 (0.65–0.66)
**Work Environment**		
Indoor		
Mix Indoor and Outdoor	0.75 (0.27–2.05)	0.77 (0.72–0.81)
Outdoor	0.92 (0.18–4.25)	1.25 (1.15–1.29)
Students, retired, unemployed	1.02 (0.39–2.62)	1.14 (1.13–1.15)
**Educational level**		
Secondary		
Primary or none	1.61 (0.65–4.27)	1.31 (1.29–1.32)
**Freshwater exposure**		
No		
Yes	**13.33 (1.20-166.58)**	6.51 (5.95–6.99)
**Living in a flooding-risk area**		
No		
Yes	1.14 (0.50–2.70)	0.95 (0.89–1.06)
**Socio-economic drivers**		
Motorised time to health unity (IQR, time in minutes)		
0.00–1.37	Ref	Ref
1.38–2.85	1.31 (0.25–7.33)	0.85 (0.82–0.87)
2.86–4.68	0.97 (0.18–5.55)	0.67 (0.64–0.72)
4.69–11.32	2.43 (0.38–17.95)	1.15 (1.13–1.19)
GDP^1^	0.66 (0.32–1.28)	0.73 (0.72–0.74)
Bare ground percentage (% of area coverage)		
< 1.44% area coverage	Ref	Ref
≥ 1.44% area coverage	9.24 (0.87-102.99)	3.70 (3.49–3.92)
Cropland percentage (%of area coverage)		
0.00–0.00	Ref	Ref
0.00–3.85	2.52 (0.54–12.46)	1.86 (1.69–2.01)
3.86–42.15	1.20 (0.23–6.27)	1.20 (1.08–1.28)
42.16–87.85	1.88 (0.32–11.20)	1.44 (1.31–1.51)
River density (total metres)		
0.00–0.00	Ref	
0.00–235.6	0.47 (0.02–7.13)	0.54 (0.45–0.65)
235.7–1048.0	**6.78 (1.94–29.51)**	2.43 (2.41–2.45)
Average precipitation (5-y average in mm)		
80.00–85.50	Ref	Ref
85.51–86.59	3.62 (0.84-18)	2.53 (2.42–2.66)
86.59–88.68	2.58 (0.55–12.75)	1.81 (1.76–1.84)
88.69–95.14	**5.41 (1.39–24.81)**	2.57 (2.52–2.65)

^1^Per 1,000,000 USD increase in the GDP.

**Fig. 2:**
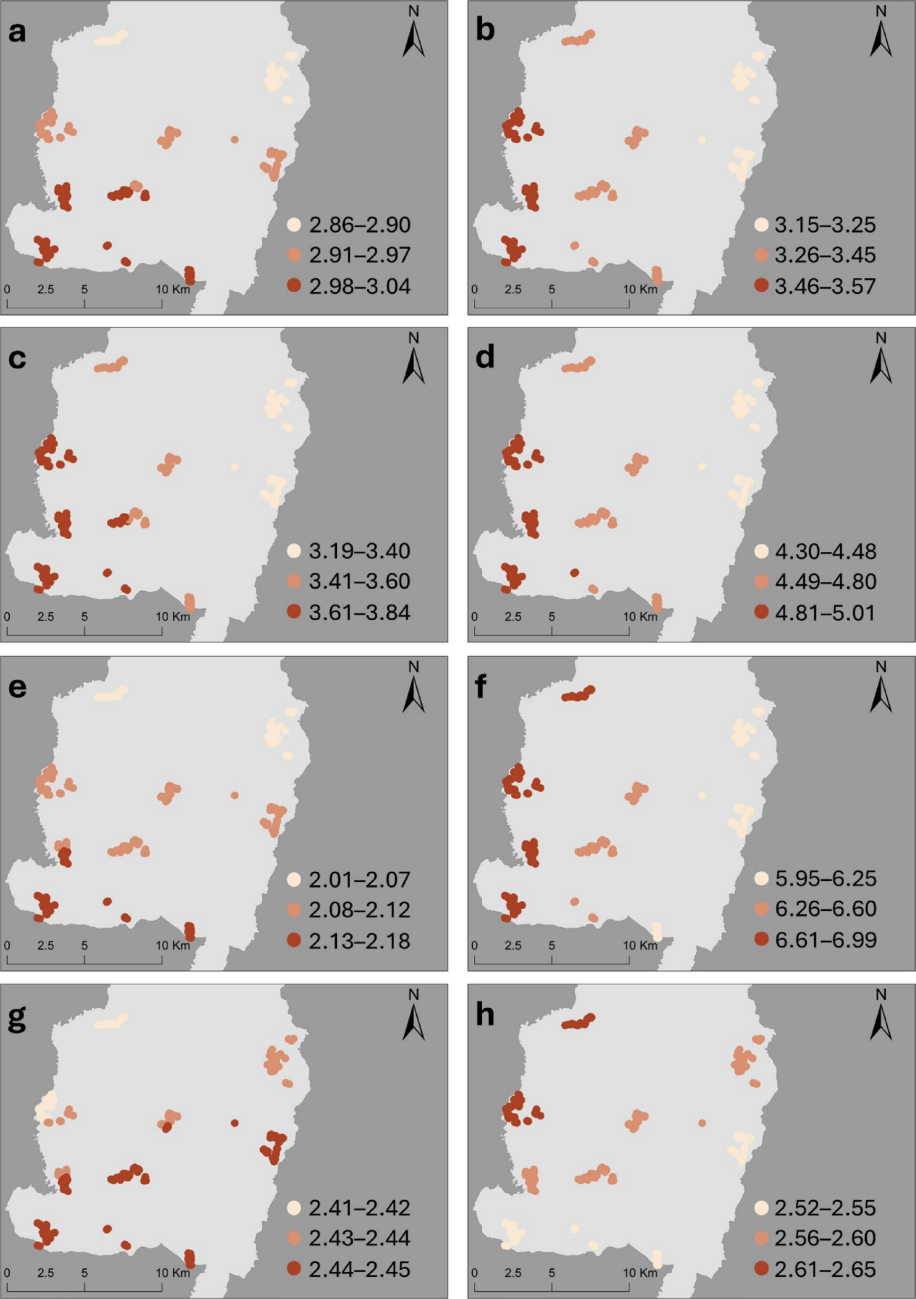
Spatial variation in odd ratios for leptospirosis seropositivity from geographically weighted regression, Espaillat Province. a-d: Age groups [a) 20–34 years, b) 35–49 years, c) 50–64 years, d) ≥ 65 years], e) gender male, f) exposure to freshwater, g) the percentage of bare ground in a 250 m buffer around the household, h) total river length in a 250 m buffer around the household. Each dot represents a surveyed household, and colours represent OR at the household location for each covariate. Maps were created in Esri^®^ ArcGIS software v 10.8 (https://www.esri.com/en-us/arcgis/products/arcgis-desktop/resources, Esri^®^ ArcMap 10.8.0.12790. Redlands, CA, USA)^13^, base layer from layer from United Nations OCHA – COD-AB dataset (https://data.humdata.org/dataset/cod-ab-dom), licensed under CC BY-IGO.

Results for Espaillat Province GGWR model are presented in Fig. 2, showing the spatial variation in OR of variables significantly associated with leptospirosis seropositivity in the province-specific GLMER model. In the GGWR model, the widest range of variation of leptospirosis seropositivity OR across the province was associated with freshwater exposure (median OR 6.51, ranging from 5.94 to 6.98 across the study areas). In contrast, the OR associated withethinic group mullato had the lowest variation, ranging from 0.65 to 0.66 (median 0.66).

### San Pedro de Macoris

In the multivariable GLMER, variables associated with significantly higher OR of leptospirosis seropositivity included older age groups (reference 5–19 years), namely: 20–34 years OR 4.90 (95%CI 1.65–16.20); 35–49 years OR 9.33 (2.95-35.07); 50–64 years (OR 6.51;2.02–25.76); and ≥ 65 years OR 12.65 (3.64–57.48). Male gender compared to female (OR 4.72;2.43–10.65)  and exposure to rats compared to no exposure (OR 2.85;1.16–7.75) were also significant risk factors in this province (Table 3).

**Table 3:** Odds ratios (ORs) and 95% CI from the generalised linear mixed-effects regression (GLMER) and OR median and range from the geographically weighted regression for leptospirosis seropositivity in San Pedro de Macoris province, Dominican republic, 2021.

	GLMER OR (95%CI)	GGWR Median (Min, Max)	
Age (years)			
5–19	Ref	Ref	
20–34	**4.90 (1.65–16.20)**	4.70 (2.74–5.18)	
35–49	**9.33 (2.95–35.07)**	7.44 (4.62–7.65)	
50–64	**6.51 (2.02–25.76)**	5.94 (3.71–6.30)	
≥ 65	**12.65 (3.64–57.48)**	7.81 (5.71–8.38)	
**Gender**			
Female	Ref	Ref	
Male	**4.72 (2.43–10.65)**	3.34 (2.82–3.41)	
Other	1.76 (0.08–24.36)	2.03 (1.63–4.72)	
**Ethnic group**			
Indigenous	Ref	Ref	
Mestizo	0.83 (0.33–2.13)	1.08 (0.86–1.09)	
Mulatto	1.09 (0.44–2.79)	1.21 (0.87–1.29)	
**Work Environment**			
Indoors	Ref	Ref	
Mix Indoor and Outdoor	2.23 (0.83–6.46)	1.30 (1.24–2.81)	
Outdoor	1.88 (0.51–7.40)	0.85 (0.65–3.38)	
Students, retired, unemployed	1.76 (0.81–4.04)	1.53 (1.23–1.55)	
**Educational level**			
Secondary	Ref	Ref	
Primary or none	1.36 (0.67–2.76)	1.10 (1.07–1.41)	
**Freshwater exposure**			
No	Ref	Ref	
Yes	0.76 (0.28–1.99)	0.82 (0.72–0.92)	
**Living in a flooding-risk area**			
No	Ref	Ref	
Yes	2.66 (0.96–7.43)	3.65 (0.94–3.94)	
**Rat exposure**			
No	Ref	Ref	
Yes	**2.85 (1.16–7.75)**	2.59 (1.23–3.14)	
**Socio-economic drivers**			
Motorized time to health unity (in minutes)			
0.00–1.57	Ref	Ref	
1.58–3.40	1.38 (0.41–5.01)	0.87 (0.79–2.47)	
3.41–7.15	1.98 (0.34–11.46)	1.58 (1.01–1.83)	
7.16–18.78	3.77 (0.63–25.21)	2.44 (2.33–3.83)	
GDP (1,000,000 USD)			
0.03–2.08	Ref	Ref	
2.09–5.64	1.72 (0.47–7.06)	1.65 (0.49–1.71)	
5.65–14.20	0.55 (0.09–2.9)	0.62 (0.17–0.67)	
14.21–40.96	1.22 (0.26–5.88)	1.08 (0.45–1.16)	
**Environmental drivers**			
Cropland percentage^1^	0.96 (0.51–1.76)	0.98 (0.79–0.98)	
River density			
> 250 m	Ref	Ref	
≥ 250 m	0.97 (0.20–4.55)	0.52 (0.42–3.80)	
Average precipitation^2^	0.71 (0.25–1.98)	0.79 (0.70–0.85)	

^1^Per each 1% increase in the land cover ground surrounding the household. ^2^Per 1 mm increase in the 5-y average rainfall.

**Fig. 3:**
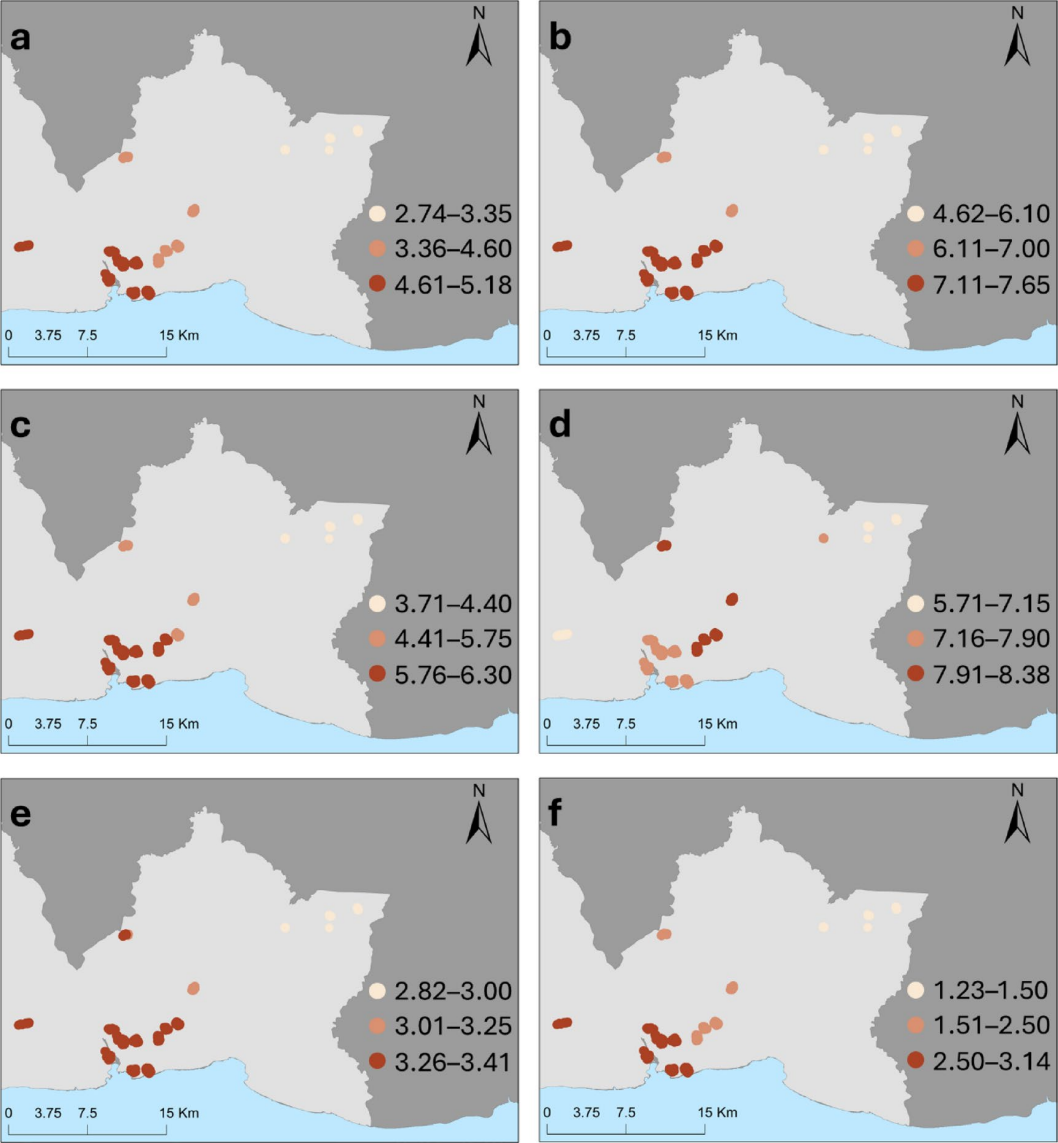
Spatial variation in odd ratios for leptospirosis from geographically weighted regression, San Pedro de Macoris Province. a-d: Age groups [a) 20–34 years, b) 35–49 years, c) 50–64 years, d) ≥ 65 years], e) gender male, f) exposure to rats. Each dot represents a surveyed household, and colours represent odds ratios at the household location for each covariate. Maps were created in Esri^®^ ArcGIS software v 10.8 (https://www.esri.com/en-us/arcgis/products/arcgis-desktop/resources, Esri^®^ ArcMap 10.8.0.12790. Redlands, CA, USA)^13^, base layer from layer from United Nations OCHA – COD-AB dataset (https://data.humdata.org/dataset/cod-ab-dom), licensed under CC BY-IGO.

Results from the SPM Province GGWR model are presented in Fig. 3, showing the spatial variation in OR of variables significantly associated with leptospirosis seropositivity in the province-specific GLMER model. In the GGWR model for SPM, OR of leptospirosis seropositivity associated with river density within a 250-meter buffer surrounding the household above 250m length exhibited the widest variation across the province (median OR 0.52; ranging from 0.42 to3.80). In contrast, the OR associated with average precipitation in the last five yeras had the lowest variation, ranging from 0.70 to 0.85 (median 0.79).

## Discussion

Our study identified considerable spatial variation in the sociodemographic and environmental drivers of leptospirosis seropositivity within and between the two provinces investigated in the DR, requiring the construction of specific models for each province. Despite this variation, older age groups and male gender were associated with higher odds of leptospirosis seropositivity across both provinces, in accordance with previously reported higher burden of disease among males in the Caribbean^15,16^ and globally^17^. While there was some overlap in the variables included in the final province-specific GGWR models, there were crucial differences in the final set of variables and their association with leptospirosis seropositivity. The importance of risk factors frequently associated with leptospirosis such as freshwater and rat exposure, and outdoor work environment^16,18^, varied substantially between the two provinces, illustrating the important contribution that spatial analyses can make for informing more targeted and precise public health interventions^19^. In this sense, while in Espaillat effectiveness of public health interventions could benefit from focusing on guidance regarding contact with freshwater, in SPM measures to reduce and control rat population (e.g.: waste management) would have greater impact. The final models for each province included different sets of variables as well as different definitions of categories for some of variables, such as varying buffer sizes and aggregation strategies. For example, river density was extracted using a 250 m buffer in Espaillat, while a 500 m buffer was used in SPM, reflecting differences in the spatial scale at which environmental drivers demonstrated the strongest correlation with transmission risk. Additionally, some continuous variables were aggregated differently across provinces to account for non-linear relationships, such as categorisation based on quartile distributions, further tailoring the models to local data characteristics. While these differences limit direct comparisons of each driver between provinces, they enhance the ability to detect context-specific drivers of leptospirosis, supporting more precise and locally relevant public health interventions.

Leptospirosis is traditionally considered an occupational disease^20^, and young males are especially affected in resource-limited rural areas^21^ where work-related activities, such as animal husbandry and agriculture, take place in outdoor environments^4,20,22^. However, in our study, the association between leptospirosis seropositivity and outdoor work environment was not significant in the GLMER model for both provinces. The GGWR models indicated differences between the two provinces, with increased OR of leptospirosis seropositivity associated with outdoor work environments in Espaillat but not in SPM. This could be due to the predominance of farm-related activities in the former^23^. While leptospirosis seroprevalence studies typically report a peak in prevalence in young and middle-aged adults followed by a decrease in older age groups^17^, our results diverge from these findings. In Espaillat, the GGWR revealed a continuous rise in OR across age groups, while in SPM, two peaks were reported (35–49 and ≥ 65 years) indicating a complex age-specific risk profile in the DR. Partially, this unique profile could be explained by the association of recurrent exposures throughout life and antibodies lasting long periods^24^ with slower decay after repeated infections^1^. However, these two factors are not unique to the DR, thus suggesting sustained exposure and transmission in older age groups.

Water plays a crucial role in the transmission cycle of leptospirosis, with pathogenic *Leptospira* capable of persisting in moist soil and freshwater for extended periods^25^. Heavy rainfall, cyclones, and flooding events have been associated with leptospirosis outbreaks in many different environmental settings around the world^1,18^. Studies show that floods, cyclones and extreme rainfall events might become more frequent as the world becomes warmer, creating more favourable conditions for leptospirosis transmission. In addition to traditionally recognised high-risk freshwater exposure, there is growing evidence that recreational exposure to previously considered low-risk freshwater (e.g., waterfalls and rivers) during sports such as triathlon, kayaking, and whitewater rafting can also contribute to outbreaks [ref], highlighting the multifaceted nature of water-related risk. In this context, unpacking spatial variation of the importance of specific drivers could be fundamental to the success of targeted public health interventions. In Espaillat, results from the GGWR identified freshwater exposure as an important risk factor, and other water-related variables, such as river density and average precipitation in the last five years, were associated with increased OR across this province. However, in SPM, water-related variables were not associated with leptospirosis seroprevalence. Differences in urbanisation levels and primary economic activities might have impacted the relative importance of determinants between provinces. In Espaillat, additionally to having a larger proportion of population living in rural areas compared to SPM (54.7% and 5.9%, respectively), animal husbandry is the main farming activity, while in SPM agricultural practices is distributed across animal husbandry and crop production. Recent studies conducted in slum settlements in Latin America found no evidence of the association between flooding and other water exposure and leptospirosis cases^16,26^, suggesting that the impact of water-related events on leptospirosis prevalence might be non-linear and vary between specific contexts. In urban settings, leptospirosis transmission is mostly associated with poor sanitation, proximity to sewage, solid waste collection, and an increased rat population^16,18^. In our study, rat exposure exhibited a strong positive association with seropositivity in SPM but not in Espaillat. In the latter, the absence of seropositive participants who reported positive exposure limited the inclusion of this covariate in the final province-specific model. Leptospirosis is highly associated with poverty in rural and urban settings^2,17^. In Espaillat, a higher GDP at the community-level was associated with lower OR of leptospirosis seropositivity, suggesting that poverty might be an important determinant of infection. In SPM, the nonlinear association between GDP and leptospirosis seropositivity required the analysis to be conducted by aggregating GDP in groups based on quartile distribution. Yet, no quartile was significantly associated with leptospirosis seropositivity.

While our findings offer valuable insights into the spatial dynamics of leptospirosis transmission, certain aspects of the study design and data availability inevitably shaped the scope of our conclusions. First, due to the cross-sectional design of this study, temporal patterns and trends could not be assessed; therefore, our study might not reflect any recent epidemiological changes in the transmission patterns. Second, the analysis was restricted to only two of the 31 provinces plus Santo Domingo National District. As our results show, leptospirosis drivers and risk factors vary across space, limiting the generalisation of our findings throughout the country and the Caribbean region. Third, some questionnaire variables could also have benefited from greater detail. The design of a household survey is always a balance between the level of detail we would like to have, and the number and complexity of questions that a field team can reasonably be expected to ask each participant. For instance, rat exposure was recorded as a binary variable, without capturing frequency or intensity, which may have limited our ability to detect more nuanced associations. Similarly, while freshwater exposure included a comprehensive characterisation of the type of exposure, the sample size may have constrained our ability to fully explore its relationship with seropositivity. Additionally, the questionnaire collected self-reported ethnicity, yet the interpretation of this results can be complex, especially in countries with multiple heritages. It is important to notice that there is growing evidence associating socially assigned race and health outcomes through discrimination and socioeconomic status^27^ and showing that incomplete reporting of ethnic groups and race can limit actions on reducing inequalities^28^. In this study, we used a robust variable selection procedure, in which this variable was selected for the final model. Nevertheless, results from the final model did not identify significant differences in leptospirosis seropositivity and ethnic groups. Fourth, our analysis was conducted by aggregating all serogroups, but transmission pathways, reservoirs mammals, and risk factors might differ between serogroups. Combining serogroups for our analyses might have obscured specific risk factors, which can be crucial for targeted public health interventions. Fifth, environmental variables included in this study were limited by publicly available data. Important risk factors such as farm animal density and proximity to sewage^22^ were not included, as data were mostly not available, or when available, the spatial resolution was limited to the province level and not suitable for our analysis. This limitation might have impacted model performance differently between the two provinces. In SPM, besides older age groups and male gender, exposure to rats was the only variable significantly associated with leptospirosis seropositivity in the GLMER models, suggesting the existence of relevant risk factors and drivers in this province that were not captured by our model. Finally, differences in variable selection and representation between the province-specific models—such as buffer sizes and categorisation of continuous variables—also limit direct comparisons. However, this tailored approach allowed us to identify highly localised risk factors, which are essential for informing context-specific public health strategies. To ensure the inclusion of relevant variables in each province, we searched multiple data sources to obtain a comprehensive dataset of climatic, environmental and sociodemographic factors that can be spatially linked to our survey data. One of the strengths of our study is the detailed data extraction process; for most of the spatially linked variables, we explored multiple approaches to extract the data. Our analysis provided individual and household-level information regarding risk factors and drivers associated with leptospirosis transmission, identifying variation of transmission patterns on a fine spatial scale.

Our results contribute to a better understanding of leptospirosis epidemiology in the DR. Similarly to studies conducted in South-East Asia and Western Pacific regions we unveil the variation in the importance of local drivers of leptospirosis transmission^29,30^. By doing so, this research highlights the need for tailored public health interventions that can vary on a fine spatial scale. Effective control measures must adapt to the specific risk factors in each province and community, prioritising different strategies based on local conditions. For instance, some communities may benefit from interventions focusing on reducing freshwater exposure, while others may benefit from controlling rat populations. The success of public health actions depends on knowing which factors most significantly impact each community, enabling more informed, efficient and impactful decision-making.

## Supplementary Information

Below is the link to the electronic supplementary material.

Supplementary Material 1

## Data Availability

A de-identified dataset is available at https://github.com/enilles1/DR-Leptospirosis for the purpose of reproducing and building on the analyses.
