# Supplementary material for: Quantifying Spatial variation in environmental and sociodemographic drivers of leptospirosis in the Dominican Republic using a geographically weighted regression model
Source: Sci Rep. 2025 Jul 25;15:27073. doi: 10.1038/s41598-025-13413-5 (PMC12297537; doi:10.1038/s41598-025-13413-5)
Supplement: Supplementary file 1 — Supplementary Material 1 [file 41598_2025_13413_MOESM1_ESM.docx]

***Supplementary material***

**Quantifying spatial Variation in environmental and sociodemographic drivers of leptospirosis in the Dominican Republic using a geographically weighted regression model**

Contents

[Methods: 2](#_Toc196297100)

[Survey design 2](#_Toc196297101)

[Survey data collected and included in the preliminary analysis. 2](#_Toc196297102)

[Spatial data considered for the analysis. 4](#_Toc196297103)

[Data Source 4](#_Toc196297104)

[Data extraction: 6](#_Toc196297105)

[Climate graphs: 7](#_Toc196297106)

[Bare ground distribution in the DR 9](#_Toc196297107)

[Climate, environment and sociodemographic characteristics of Espaillat and San Pedro de Macoris. 10](#_Toc196297108)

[Preliminary models results: 11](#_Toc196297109)

[Bivariate analysis: 11](#_Toc196297110)

[Models Construction 13](#_Toc196297111)

[Non-spatial analysis 13](#_Toc196297112)

[Spatial analysis 13](#_Toc196297113)

[Final set of variables included in the province-specific models 15](#_Toc196297114)

[Models’ performance 16](#_Toc196297115)

[References: 17](#_Toc196297116)

# Methods:

## Survey design

Between 30 June and 12 October 2021, a three-stage cross-sectional national serosurvey was conducted in the DR. Detailed descriptions of the survey design and data collection have been previously reported (1). In summary, the 31 provinces in the DR, plus Santo Domingo National District were divided into five regions. Based on population size and distribution between rural and urban settings, a predefined number of communities were selected from each region using a spatially representative sampling method. Household selection differed between urban and rural settings. In urban settings, selection was based on a grid method and in rural communities, based on a spatially representative sampling method that maximised spatial dispersion of sampling locations. Individual data were collected during a single household visit, and household location was recorded using GPS. This study reports on results from two provinces, Espaillat (10 communities), located in the Northwest of the country, and San Pedro de Macoris (SPM) (13 communities), located in the Southeast. These two provinces are linked to an ongoing study of clinical surveillance of acute febrile illnesses (2). In Espaillat and SPM, 60 households were selected in each community.

## Survey data collected and included in the preliminary analysis.

During the field survey, a trained fieldwork team interviewed all participants using a questionnaire. There were individual questions applied to all participants, and questions regarding the household applied only to the household representative, and their answers were linked to all members of that household In this analysis, variables I–X were derived from questions applied to each participant individually, and variables XI–XVII were derived from questions applied to the household representative and assigned to all household members.

1. Age

Collected as completed years and categorised into the following groups: *5–19 years* (reference), *2–34 years*, *35–49 years, 50–64 years* and equal or *above 65 years*.

1. Gender

*Female* (reference), *male*, *other*. Overall in both provinces, 16 participants reported gender as other, four in Espaillat and 12 in San Pedro de Macoris.

1. Country of birth/Nationality

Data was collected by two different questions:

Was the participant born in the Dominican Republic?

Categorised as a binary response (yes/no). The group of participants born in the Dominican Republic were the reference group.

Is the participant Haitian?

Categorised as a binary response (yes/no). The group of Haitian participants were the reference group.

1. Ethnic group

Categorised as *indigenous* (reference), *white*, *mestizo*, *mulatto* and *other*. *White* and *other* were combined due to the small sample size. As no participant who tested positive for leptospirosis reported being *white* in San Pedro de Macpris, we could not use this group as a reference for the analysis and results from this group are excluded in tables and figures from this province.

1. Educational level

Each participant was questioned regarding their educational level. For participants under 18 years old with missing data, the maximum educational level reported in the household was assigned. This data was categorised into two groups, *none or primary* and *secondary or tertiary or technical* (reference)*.* The remaining participants with missing data were grouped with *none or primary*.

1. Occupation

The original question included many categories. In this study, two categorizations were investigated. The first was *farmers*, *professionals (health care workers, teachers, office workers, etc; adopted as the* reference group*) and non-professionals (home care workers, shop workers, housewives/househusband, etc)*, and the second was *farmers* and *non-farmers (professionals and non-professionals; adopted as the* reference group*).*

1. Work environment

Categorised as *indoor* (reference), *outdoor*, *mixed indoor and outdoor* and *not-working* (student, retired, unemployed).

1. Informal settlement

*Investigated* if households were built on informal settlements, categorised as “yes” or “no” (*adopted as the* reference group).

1. Freshwater exposure

*Investigated* if the participant reported contact (touching, walking through, swimming, bathing, cleaning, washing) with fresh water (flood water, rivers, lakes, waterfalls), *categorised as “yes”*, or *“no”* (adopted as the reference group).

1. Animal exposure

Investigated if the participant reported contact (milking, feeding, slaughtering, trapping) with each of the following animals individually: rats or mice, pigs, sheep, goats and cows/oxen/bovine. Categorised as “yes” or “no” (adopted as reference group).

1. Number of household members

Investigated the number of people usually living in the household. Categorised as *1–2 members* and *≥3 members*.

1. City water

Investigate if the household has access to piped water (city water supply). Categorised as “yes” or “no” (adopted as reference group).

1. Toilet inside the house

Investigate if the household has a toilet inside the house. Categorised as “yes” or “no” (adopted as reference group).

1. Floor material

Investigate the materials used on the floor in the house. Original data allowed a combination of six categories. In this study, floor material was categorised as *any material* (reference) and *none/dirty*.

1. Air Conditioning

Investigate if the household has air conditioning. Categorised as “yes” or “no” (adopted as reference group).

1. Vehicle ownership

*Investigate if any of the household members owned a vehicle.* Categorised as “yes” or “no” (adopted as reference group).

1. Source of water used for hand washing at home

Categorised as *untreated* *water* (unprotected well, unprotected spring, rainwater collection, etc) and *treated water* (piped, public tap, tanker truck, etc; adopted as reference group).

## Spatial data considered for the analysis.

### Data source

Spatially referenced environmental and socioeconomic covariate layers were downloaded and extracted from the following sources and processed as indicated below:

1. *Rural-urban classification (3).* A map of the rural and urban barrio/paraje administrative boundaries was downloaded from the Dominican Republic National Statistics Office.
2. *Distance to major roads (4)*. A raster with the distance (in km) from the cell centre to the nearest major road was downloaded from the WorldPop website at a resolution of 3 arc-seconds .
3. *Distance to the provincial capital city (3).* The location of provincial capitals in the DR was extracted in a shapefile format from the Dominican Republic National Statistics Office. A raster layer of the Euclidean distance between each household location and the nearest provincial capital was generated in km (5).
4. *Distance to education facilities (6)*. Geographic locations of education facilities, including a mix of public and private institutions (kindergartens, schools, colleges and universities), were acquired from OpenStreetMap in a shapefile format. The Euclidean distance between each household and the nearest education facility was derived in km (5).
5. *Walking travel time to healthcare facilities (7)*. Estimates of travel time (in minutes) from each household to the nearest geolocated hospital or clinic were downloaded from the Malaria Atlas Project website. For locations where there was no data, a 1km buffer around the household was created, and the mean value on valid data points was extracted and assigned to the household.
6. *Motorised travel time to healthcare facilities (7)*. Estimates of travel time (in minutes) from each household to the nearest hospital or clinic by motorised transport were extracted in from the Malaria Atlas Project website. For locations where there was no data, a 1km buffer around the household was created, and the mean value on valid data points was extracted and assigned to the household.
7. *Elevation and slope (8)*. Data were obtained at a resolution of 3 arc-second from the Shuttle Radar Topographic Mission (SRTM) dataset: SRTM 2000, Dominican Republic. For elevation the value of each grid cell represents its elevation above sea level in metres (m). For locations where there was no data, a 1km buffer around the household was created, and the mean value on valid data points was extracted and assigned to the household.
8. *Land surface temperature (9)*. Satellite sensor data on land surface temperatures from the Moderate Resolution Imaging Spectroradiometer (MODIS) satellite were obtained from the USGS Earth Explorer website at 1 km resolution for every eight days from January 1 2017 to December 31 2021. The monthly average, maximum and minimum temperature for the 5 years (creating 36 variables), as well as the average, maximum and minimum of the 5 years (3 variables), was extracted at °C. For locations where there was no data, a 1km buffer around the household was created, and the mean value on valid data points was extracted and assigned to the household.
9. *Precipitation (10).* Monthly gridded rainfall time series from 2017 to 2021 was downloaded from CHIIRPS: rainfall estimates from rain gauge and satellite observations. This data set combines a 0.05° resolution satellite imagery and in-situ station data to create the rainfall grid. Data is provided in mm per month. Data extracted was aggregated to create a monthly average, maximum and minimum for the 5-year and, and 5-year average, maximum and minimum.
10. *Population density (4).* Estimates of population density for 2020 were downloaded from the WorldPop website. A raster was available for the DR at the resolution of 30 arc-seconds (approximately 1km at the equator), reported as the number of people per square kilometre (km^2^).
11. *Gross domestic product (11).* A raster with the average gross domestic product (GDP) per capita was downloaded from datadryad.org at a resolution of 5-arc-min (12). GDP is given in 2011 international US dollars. For locations where there was no data, a 1km buffer around the household was created, and the mean value on valid datapoints was extracted and assigned to the household.
12. *Socioeconomic data (3).* Socioeconomic indicators at the municipality level were obtained from the Dominican Republic National Statistics Office reports in 2021-2022. These data are projection estimates from the Census in 2010 (27). The data extracted included adult illiteracy rate (population aged >15 years), unemployment rate, number of primary healthcare units (PCU) per population (excluding hospitals, clinics and private health facilities), percentage of population without access to indoor toilet, and percentage of population with access to piped water. The values of socioeconomic variables extracted at the municipality level were assumed for the clusters in each municipality.
13. *Land use and land cover (13)*. Data were derived at 10m resolution from the Sentinel-2 Global Land Use/Land Cover (LULC) Timeseries produced by Impact Observatory, Microsoft, and the Environmental Systems Research Institute (Esri) (26). The global LULC cover map with 11 LULC classes was used to generate six separate rasters for the LULC categories that cover the DR: crops, rangelands, bare ground, trees, flooded vegetation and built/urban area (Supplementary Table 1). For each household, a 50m, 100m, 250m, 500m and 1km buffer was generated. The percentage of each LULC raster overlapping with the household buffer was extracted.
14. *River density (4)*. The total length (metres) of rivers was extracted from a vector layer of all main rivers in the DR overlapping with the 50m, 100m, 250m, 500m and 1km household buffers.
15. *Flooding-risk area.* A vector layer containing a national flooding-risk map, which delimitates areas considered to be at risk of flooding. The flooding risk-map was created by National Geologic Service (Servicio Geologico Nacional) and provided by Dr Gregorio Antonio Rosario Michel through personal communication. The flood-risk map was originally published as a figure by Michel et al (14).

**Supplementary Table 1. Land cover class definitions**

| **Land Use/Land Cover Class** | **Description** |
| --- | --- |
| **Crops** | Human planted/plotted cereals, grasses, and crops not at tree height. Examples: corn, wheat, soy, fallow plots of structured land. |
| **Rangeland** | Open areas covered in homogenous grasses with little to no taller vegetation; wild cereals and grasses with no obvious human plotting (i.e., not a plotted field). Examples: natural meadows and fields with sparse to no tree cover, open savanna with few to no trees, parks/golf courses/lawns, pastures. Mix of small clusters of plants or single plants dispersed on a landscape that shows exposed soil or rock; scrub-filled clearings within dense forests that are clearly not taller than trees; examples: moderate to sparse cover of bushes, shrubs and tufts of grass, savannas with very sparse grasses, trees or other plants. |
| **Bare ground** | Areas of rock or soil with very sparse to no vegetation for the entire year; large areas of sand and deserts with no to little vegetation; examples: exposed rock or soil, desert and sand dunes, dry salt flats/pans, dried lake beds, mines |
| **Trees** | Any significant clustering of tall (~15 feet or higher) dense vegetation, typically with a closed or dense canopy. Examples: wooded vegetation, clusters of dense tall vegetation within savannas, plantations, swamp or mangroves (dense/tall vegetation with ephemeral water or canopy too thick to detect water underneath). |
| **Built/Urban** | Human made structures; major road and rail networks; large homogenous impervious surfaces including parking structures, office buildings and residential housing. Examples: houses, dense villages / towns / cities, paved roads, asphalt. |

### Data extraction and scale:

Individual values for all spatial variables were assigned based on the location of their households. For spatial vector data, we used two extraction methods tailored to the nature of each covariate:

1. Proximity-based extraction: We calculated the straight-line distance from each household to the nearest relevant feature (e.g., schools, health facilities).
2. Buffer-based extraction: We computed the proportion of area covered by specific features (e.g., LCLU class, river density) within circular buffers of varying radii (50 m to 1 km) around each household.

For raster data, such GDP, population density, and precipitation, we extracted pixel values at the exact coordinates of each household.

These methods ensured that all environmental and socioeconomic variables were spatially referenced to the household level, enabling their use in individual-level statistical models.

We explored multiple ways to extract some environmental and sociodemographic variables. Variables such as precipitation or land use/ land cover were extracted for multiple scenarios (e.g. mean precipitation for January, February, March, …, and December between 2017 and 2021; percentage of cropland in buffers varying from 50m to 1km) to ensure optimal representation; in those cases, even when collinearity was not identified, we tested multiple multivariable models choosing one among the several variables of the same category to obtain the best performance and avoid redundancy. The same method was used to select variables related to work environment and occupation, or walking distance to and motorized travel time to health facilities.

### Climate graphs:

- - - 1. **Climate in Espaillat**

**Supplementary Figure 1. Average monthly precipitation in Espaillat province, Dominican Republic, 2017–2021.**

**Supplementary Figure 2. Average temperature in Espaillat province, Dominican Republic, 2017–2021.**

- - - 1. **Climate in San Pedro de Macoris**

**Supplementary Figure 3. Average monthly precipitation in San Pedro de Macoris province, Dominican Republic, 2017–2021.**

**Supplementary Figure 4. Average temperature in San Pedro de Macoris province, Dominican Republic, 2017–2021.**

### Bare ground distribution in the Dominican Republic

**Supplementary Figure 5. Maps of the bare ground distribution in San Pedro de Macoris province, Dominican Republic, 2021.**

**
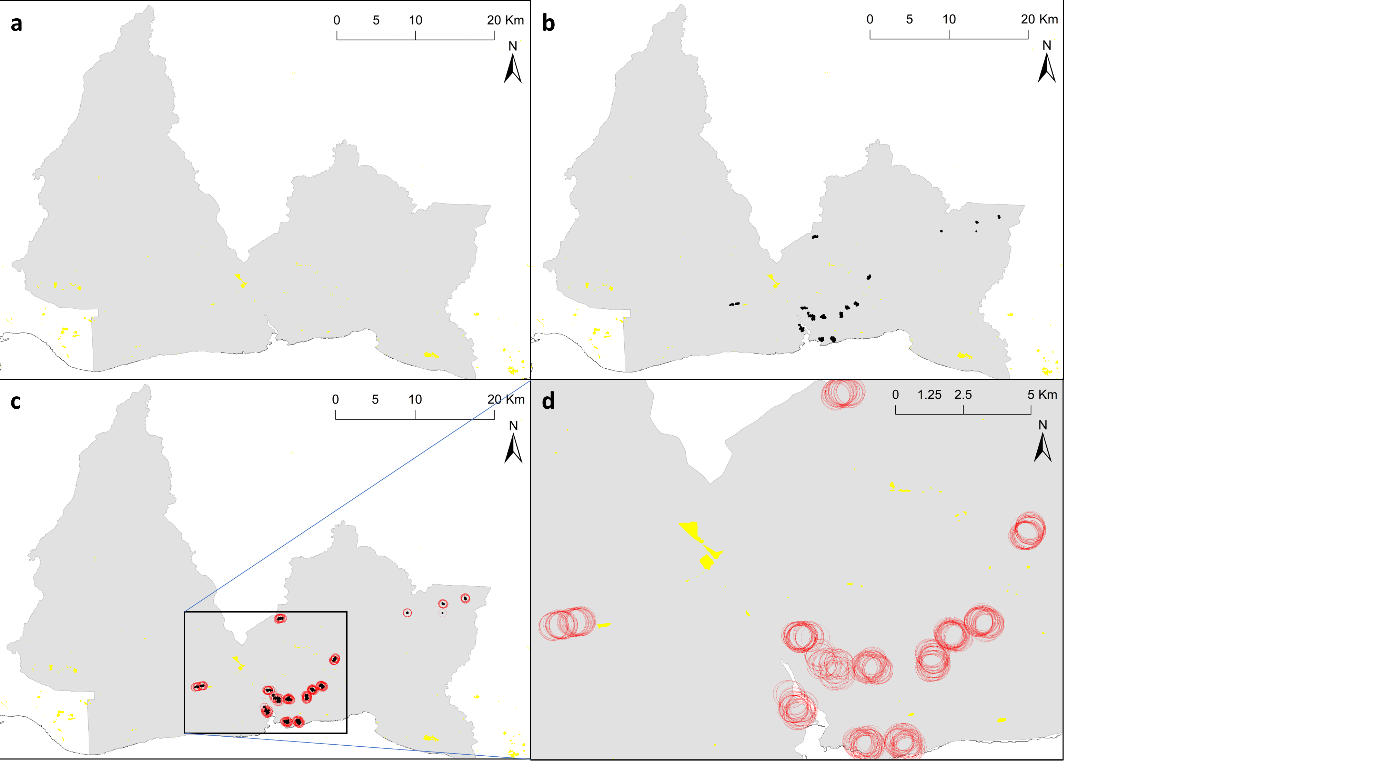
**

**a)** In yellow, bare ground distribution in San Pedro de Macoris. **b)** Black dots representing the surveyed household locations. **c)** The red circle represents a 500m buffer around each households included in the study. **d)** Zoom in, showing no overlap between the 500m buffer layer and bare ground. Maps were created in Esri® ArcGIS software v 10.8 (<https://www.esri.com/en-us/arcgis/products/arcgis-desktop/resources>, Esri® ArcMap 10.8.0.12790. Redlands, CA, USA) (5), base layer from United Nations OCHA – COD-AB dataset (<https://data.humdata.org/dataset/cod-ab-dom>) (15), licensed under CC BY-IGO.

**Supplementary Figure 6. Maps of the bare ground distribution in Espaillat province, Dominican Republic, 2021.**

**
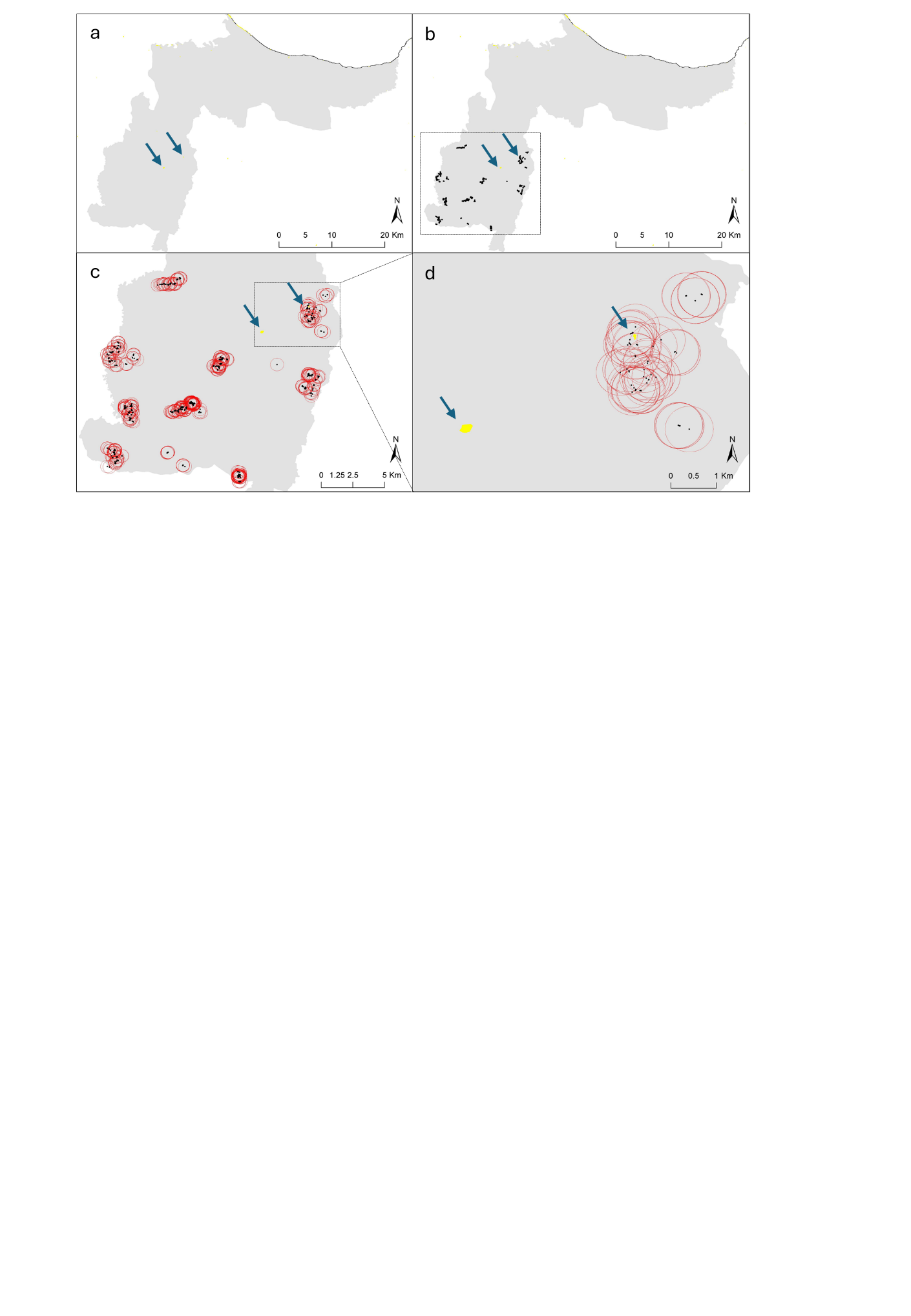
**

**a)**In yellow, bare ground distribution in Espaillat (blue arrows marking the location). **b)** Black dots representing the surveyed household locations. **c)** The red circle represents a 250m buffer around each household included in the study. **d)** Zoom in, showing no overlap between the 250m buffer layer and bare ground. Maps were created in Esri® ArcGIS software v 10.8 (<https://www.esri.com/en-us/arcgis/products/arcgis-desktop/resources>, Esri® ArcMap 10.8.0.12790. Redlands, CA, USA) (5), base layer from layer from United Nations OCHA – COD-AB dataset (<https://data.humdata.org/dataset/cod-ab-dom>) (15), licensed under CC BY-IGO.

### Climate, environment and sociodemographic characteristics of Espaillat and San Pedro de Macoris.

**Supplementary Table 2. Descriptive statistics of environmental and sociodemographic covariates extracted at the household locations, Dominican Republic.**

| **Variable** | **Combined** | **Espaillat** | **San Pedro de Macoris** |
| --- | --- | --- | --- |
| Population density | 1374·4 (18·9–8639·8) | 928·2 (40·6–7482·3) | 3828·9 (18·9–8639·8) |
| Distance to roads | 2·7 (0·1–8·9) | 2·9 (0·1–8·9) | 2·6 (0·1–6·6) |
| Distance to inland waterbodies | 0·4 (0·0–3·0) | 0·4 (0·0–3·0) | 0·4 (0·0–1·9) |
| Distance to coastline | 7·3 (0·2–44·6) | 36·6 (24·0–44·6) | 3·4 (0·2–18·4) |
| Motorized time travel to healthcare facilities | 3·4 (0·0–18·8) | 2·9 (0·0–11·3) | 3·4 (0·0–18·8) |
| Walking distance to health facilities | 37·3 (0·0–232·0) | 31·0 (0·0–135·0) | 47·0 (0·0–232·0) |
| Elevation | 30·0 (2·0–620·0) | 203·0 (131·0–620·0) | 21·0 (2·0–67·0) |
| Average rainfall in the last 5 years | 81·2 (78·5–96·7) | 87·0 (80·0–95·1) | 81·2 (78·5–96·7) |
| Slope | 2·0 (0·0–22·3) | 2·8 (0·0–22·3) | 1·6 (0·0–6·7) |
| Soil moisture | 0·2 (0·2–0·3) | 0·3 (0·3–0·3) | 0·2 (0·2–0·3) |
| Bare ground (50m buffer) | 0·0 (0·0–1·3) | 0·0 (0·0–1·3) | 0·0 (0·0–0·0) |
| Built/Urban (50m buffer) | 100·0 (0·0–100·0) | 100·0 (0·0–100·0) | 100·0 (0·0–100·0) |
| Crop area (50m buffer) | 0·0 (0·0–100·0) | 0·0 (0·0–100·0) | 0·0 (0·0–100·0) |
| Rangeland (50m buffer) | 0·0 (0·0–100·0) | 0·0 (0·0–25·1) | 0·0 (0·0–100·0) |
| Trees (50m buffer) | 0·0 (0·0–100·0) | 0·0 (0·0–100·0) | 0·0 (0·0–55·4) |
| Bare ground (100m buffer) | 0·0 (0·0–9·4) | 0·0 (0·0–9·4) | 0·0 (0·0–0·0) |
| Built/Urban (100m buffer) | 100·0 (0·0–100·0) | 94·2 (0·0–100·0) | 100·0 (0·0–100·0) |
| Crop area (100m buffer) | 0·0 (0·0–100·0) | 0·0 (0·0–95·2) | 0·0 (0·0–100·0) |
| Rangeland (100m buffer) | 0·0 (0·0–85·5) | 0·0 (0·0–32·6) | 0·0 (0·0–85·5) |
| Trees (100m buffer) | 0·0 (0·0–100·0) | 0·0 (0·0–100·0) | 0·0 (0·0–48·4) |
| Bare ground (250m buffer) | 0·0 (0·0–2·3) | 0·0 (0·0–2·3) | 0·0 (0·0–0·0) |
| Built/Urban (250m buffer) | 83·1 (0·0–100·0) | 61·4 (0·0–100·0) | 87·5 (0·0–100·0) |
| Crop area (250m buffer) | 0·0 (0·0–100·0) | 3·9 (0·0–87·8) | 0·0 (0·0–100·0) |
| Rangeland (250m buffer) | 0·0 (0·0–73·1) | 0·0 (0·0–31·9) | 0·0 (0·0–73·1) |
| Trees (250m buffer) | 0·0 (0·0–100·0) | 0·1 (0·0–100·0) | 0·0 (0·0–62·0) |
| Bare ground (500m buffer) | 0·0 (0·0–0·6) | 0·0 (0·0–0·6) | 0·0 (0·0–0·0) |
| Built/Urban (500m buffer) | 56·6 (0·0–100·0) | 50·7 (0·0–100·0) | 66·6 (0·0–100·0) |
| Crop area (500m buffer) | 1·1 (0·0–99·9) | 12·0 (0·0–88·5) | 0·0 (0·0–99·9) |
| Rangeland (500m buffer) | 1·8 (0·0–61·0) | 1·7 (0·0–35·8) | 2·1 (0·0–61·0) |
| Trees (500m buffer) | 2·1 (0·0–100) | 3·7 (0·0–100) | 0·4 (0·0–60·4) |
| Bare ground (1km buffer) | 0·0 (0·0–1·5) | 0·0 (0·0–0·1) | 0·0 (0·0–1·5) |
| Built/Urban (1km buffer) | 56·6 (0·0–100) | 50·7 (0·0–100) | 66·6 (0·0–100·0) |
| Crop area (1km buffer) | 4·2 (0·0–99) | 21·5 (0·0–81·6) | 0·3 (0·0–99·0) |
| Rangeland (1km buffer) | 6·2 (0·0–42·5) | 5·5 (0·0–29·2) | 7·5 (0·0–42·5) |
| Trees *(1km buffer) | 7·9 (0·0–98·6) | 9·0 (0·0–98·6) | 6·8 (0·0–63·5) |
| Flooding-risk area | 115·0 (57·0–217·0) | 165·0 (75·0–217·0) | 90·0 (57·0–129·0) |
| Maximum educational level at the household | 4·0 (1·0–6·0) | 4·0 (1·0–6·0) | 4·0 (1·0–6·0) |
| Distance to province capital | 5·5 (0·1–23·8) | 5·7 (0·1–11·1) | 4·5 (1·0–23·8) |
| Distance to educational facilities | 1·9 (0·1–20·2) | 1·5 (0·1–6·3) | 2·7 (0·2–20·2) |
| NDVI (maximum) | 0·8 (0·7–0·9) | 0·8 (0·7–0·9) | 0·8 (0·7–0·8) |
| GDP | 5·2 (0·0–46·5) | 4·6 (0·1–46·5) | 5·7 (0·0–41·0) |
| River density (250m buffer) | 0·0 (0·0–1048·0) | 0·0 (0·0–1048·0) | 0·0 (0·0–467·1) |
| River density (500m buffer) | 0·0 (0·0–3050·9) | 0·0 (0·0–3050·9) | 1. (0·0–1059·7) |

# Preliminary models results:

## Bivariate analysis:

**Supplementary Table 3. Results from bivariate logistic regression for leptospirosis seropositivity in the Dominican Republic, 2021.**

| **Covariate** | **p-value*** | | |
| --- | --- | --- | --- |
|  | **Combined** | **Espaillat** | **San Pedro de Macoris** |
| Population density | 0·00 | 0·39 | 0·00 |
| Distance to roads | 0·66 | 0·06 | 0·25 |
| Distance to inland waterbodies | 0·00 | 0·05 | 0·00 |
| River density (250m buffer) | 0·00 | 0·06 | 0·99 |
| River density (500m buffer) | 0·01 | 0·28 | 0·18 |
| Motorized time travel to healthcare facilities | 0·03 | 0·05 | 0·01 |
| Walking distance to health facilities | 0·10 | 0·17 | 0·00 |
| GDP | 0·00 | 0·12 | 0·02 |
| Elevation | 0·00 | 0·80 | 0·00 |
| Distance to educational facilities | 0·76 | 0·09 | 0·01 |
| Distance to province capital | 0·02 | 0·27 | 0·01 |
| Bare ground (50m buffer) | 0·03 | 0·05 | 0·46 |
| Built/Urban (50m buffer) | 0·13 | 0·71 | 0·00 |
| Crop area (50m buffer) | 0·09 | 0·20 | 0·99 |
| Rangeland (50m buffer) | 0·73 | 0·88 | 0·39 |
| Trees (50m buffer) | 0·61 | 0·56 | 0·55 |
| Bare ground (100m buffer) | 0·07 | 0·14 | 0·31 |
| Built/Urban (100m buffer) | 0·02 | 0·01 | 0·00 |
| Crop area (100m buffer) | 0·01 | 0·14 | 0·31 |
| Rangeland (100m buffer) | 0·67 | 0·86 | 0·34 |
| Trees (100m buffer) | 0·55 | 0·78 | 0·73 |
| Bare ground (250m buffer) | 0·04 | 0·13 | 0·17 |
| Built/Urban (250m buffer) | 0·00 | 0·04 | 0·00 |
| Crop area (250m buffer) | 0·00 | 0·07 | 0·07 |
| Rangeland (250m buffer) | 0·49 | 0·42 | 0·05 |
| Trees (250m buffer) | 0·44 | 0·48 | 0·16 |
| Bare ground (500m buffer) | 0·08 | 0·27 | 0·17 |
| Built/Urban (500m buffer) | 0·01 | 0·13 | 0·00 |
| Crop area (500m buffer) | 0·02 | 0·26 | 0·09 |
| Rangeland (500m buffer) | 0·75 | 0·23 | 0·21 |
| Trees (500m buffer) | 0·31 | 0·48 | 0·23 |
| Bare ground (1km buffer) | 0·70 | 0·7 | 0·17 |
| Built/Urban (1km buffer) | 0·01 | 0·13 | 0·00 |
| Crop area (1km buffer) | 0·03 | 0·34 | 0·10 |
| Rangeland (1km buffer) | 0·83 | 0·69 | 0·18 |
| Trees (1km buffer) | 0·27 | 0·65 | 0·61 |
| Slope | 0·00 | 0·18 | 0·74 |
| Soil moisture | 0·00 | 0·05 | 0·01 |
| Flooding risk | 0·00 | 0·27 | 0·10 |
| Average rainfall in the last 5 years | 0·00 | 0·00 | 0·08 |
| Maximum rainfall in the last 5 years | 0·29 | 0·00 | 0·01 |
| NDVI (maximum) | 0·09 | 0·51 | 0·35 |
| Age (20–34 years) | 0·00 | 0·05 | 0·00 |
| Age (35–49 years) | 0·00 | 0·02 | 0·00 |
| Age (50–64 years) | 0·00 | 0·01 | 0·00 |
| Age (≥65 years) | 0·00 | 0·00 | 0·00 |
| Gender (Male) | 0·00 | 0·00 | 0·00 |
| Gender (Other) | 0·14 | 0·13 | 0·55 |
| Setting (Rural) | 0·00 | 0·09 | 0·21 |
| Informal settlement (yes) | 0·42 | 0·34 | 0·38 |
| Number of household members <3 (yes) | 0·00 | 0·05 | 0·07 |
| *Access to piped water (no)* | 0·92 | 0·73 | 0·55 |
| Home toilet inside the house (no) | 0·32 | 0·90 | 0·12 |
| Floor material (anything but dirty) | 0·14 | 0·43 | 0·99 |
| *Air conditioning at home (no)* | 0·61 | 0·98 | 0·75 |
| *Vehicle ownership (no)* | 0·88 | 0·75 | 0·22 |
| *Drinking water source treated/piped (no)* | 0·71 | 0·79 | 0·8 |
| *Born in the DR (no)* | 0·83 | 0·51 | 0·49 |
| *Born in Haiti (yes)* | 0·47 | 0·82 | 0·25 |
| White or other | 0·58 | 0·34 | 0·88 |
| Mestizo | 0·10 | 0·19 | 0·21 |
| Mulatto | 0·96 | 0·99 | 0·99 |
| White or other | 0·47 | 0·17 | 1·00 |
| Primary or none | 0·00 | 0·03 | 0·00 |
| Farmer (yes) | 0·00 | 0·00 | 0·00 |
| Freshwater exposure (yes) | 0·85 | 0·12 | 0·63 |
| Contact with rats (yes) | 0·45 | 0·99 | 0·01 |

**In blue, p-values <0.05.*

## Models Construction

### Non-spatial analysis

The multivariate mixed-effect model for both provinces included cluster as the random effect.

For Espaillat province, fixed effects were age groups, gender, work environment, educational level, exposure to freshwater, household located in a flooding-risk area, motorized travel time to health facilities, GDP, percentage of bare ground and cropland occupying a 250m buffer around each household, total river length in a 250m buffer around each household and 5-year average precipitation as fixed effects.

For San Pedro de Macoris province, fixed effects were age groups, gender, work environment, educational level, exposure to freshwater, exposure to rats, household located in a flooding-risk area, motorized travel time to health facilities, GDP, percentage of cropland occupying a 250m buffer around each household, total river density in a 500m buffer around each household and 5-year average precipitation as fixed effects.

The same set of variables for each province was adopted in the GWR models.

### Spatial analysis

For each province model, a fixed bandwidth was selected, and both adopted bisquare weighting functions. A generalised GWR model produces multiple coefficients of variation and $\beta$ coefficients, one for each observation in the sample, and can be expressed formally as:

$$\boldsymbol{Ln(}\frac{\boldsymbol{Pi}}{\boldsymbol{1-Pi}}\boldsymbol{)=}\sum_{\boldsymbol{k}} \boldsymbol{\beta}_{\boldsymbol{k}}\boldsymbol{(}{\boldsymbol{u}_{\boldsymbol{i}}\boldsymbol{v}}_{\boldsymbol{i}}\boldsymbol{)}\boldsymbol{x}_{\boldsymbol{ki}}\boldsymbol{+\varepsilon}_{\boldsymbol{i}}$$

Where:

$$\boldsymbol{Ln(}\frac{\boldsymbol{Pi}}{\boldsymbol{1-Pi}}\boldsymbol{)}$$

is the predicted odds for the $i$th observation and $x_{ki}$ and $\varepsilon_{i}$ are, respectively, the $k$th covariate and error at the observation $i$; $\boldsymbol{(}{\boldsymbol{u}_{\boldsymbol{i}}\boldsymbol{v}}_{\boldsymbol{i}}\boldsymbol{)}$ is the x,y location of the $i$th observation; and $\beta_{k}(u_{i}v_{i})$ the coefficient for the $k$th independent variable for the observation at the location $i$. The results of the odds ratio (OR) for each variable are reported showing the median, minimum and maximum value in each province highlighting variation throughout the space.

The final variables included in the model are represented in the function below (variables marked with (*) vary between province specific-models).

seropos ~ AgeGroup + Gender + RiverDensity(250m)^*^ + MotorizedTimeTravel +

GDP + BareGround(250m) + CropLand(250m)^*^ + Setting+

FloodingRisk + Pr5Avg + HHMembers +

EthnicGroup + Occupation + SoilMoisture+

FreshwaterExp + AnimalContact^*^ + MaxEducationalLevel

After conducting the generalised bivariate mixed-effect regression and checking for collinearity (Supplementary Tables 4-6), questionnaire data included in the analysis were gender (female, male and other), age group (5–19, 20–34, 35–49, 50–64 and ≥65 years old), work environment (indoor, outdoor, mix of indoor and outdoor environment, and currently not working [such as students, unemployed, retired, other]), educational level (none or primary or secondary, and tertiary or technical), ethnic group (indigenous, mulatto, mestizo, white or other), freshwater exposure (yes/no), and exposure to rats (yes/no). In Espaillat, no participant reported exposure to rats among seropositive participants, thus this variable was excluded from the final models for this province. Similarly, in San Pedro de Macoris, none of the participants who reported the ethnic group white was seropositive for leptospirosis. In this case, only the ethnic group white was omitted from the results of this province.

Spatial environmental and sociodemographic data included in this analysis were households located in a flooding-risk area (yes/no), river density (total length of inland water bodies within a 250m (for Espaillat province) and 500m (for San Pedro de Macoris province) buffer from each household), motorized time to health facilities, gross domestic product (GDP), percentage of the 250m buffer around the household occupied by cropland and bare ground, mean rainfall volume (mm) during previous 5 years (2017-2021) (Table 1). When adopting a 250m buffer around the households, both bare ground percentage and river length were zero for all participants in San Pedro de Macoris province. Increasing the buffer size to 500m was able to capture river length in some locations, but no buffer size captured bare ground, as this land cover is uncommon in San Pedro de Macoris.

**Supplementary Table 4. Results from the Variance Inflation Factor (VIF) from the multivariable model for the combined data set, Dominican Republic, 2021.**

| **Variable** | **VIF** | **95%CI** |
| --- | --- | --- |
| Age | 1·49 | (1·41–1·58) |
| Gender | 1·34 | (1·28–1·42) |
| River density | 1·85 | (1·74–1·97) |
| Motorized travel time to health facilities | 3·11 | (2·9–3·35) |
| GDP | 1·42 | (1·35–1·51) |
| Bare ground | 1·18 | (1·13–1·25) |
| Crop area | 1·55 | (1·47–1·65) |
| Flooding risk | 1·44 | (1·37–1·53) |
| Rainfall | 2·92 | (2·72–3·13) |
| Ethnicity | 1·31 | (1·25–1·39) |
| Work environment | 1·60 | (1·52–1·7) |
| Exposure to freshwater | 1·43 | (1·36–1·51) |
| Contact with rat | 1·68 | (1·59–1·79) |
| Educational level | 1·18 | (1·13–1·25) |

**Supplementary Table 5. Results from the Variance Inflation Factor (VIF) from the multivariable model for the Espaillat data set, Dominican Republic, 2021.**

| **Variable** | **VIF** | **95%CI** |
| --- | --- | --- |
| Age | 1·76 | (1·61–1·95) |
| Gender | 1·46 | (1·35–1·61) |
| River density | 2·27 | (2·05–2·53) |
| Motorized travel time to health facilities | 1·95 | (1·77–2·17) |
| GDP | 1·73 | (1·58–1·91) |
| Bare ground | 1·31 | (1·22–1·44) |
| Crop area | 1·61 | (1·48–1·78) |
| Flooding risk | 1·51 | (1·39–1·67) |
| Rainfall | 1·73 | (1·59–1·92) |
| Ethnicity | 1·31 | (1·22–1·44) |
| Work environment | 1·72 | (1·58–1·91) |
| Exposure to freshwater | 1·15 | (1·09–1·27) |
| Educational level | 1·25 | (1·17–1·37) |

**Supplementary Table 6. Results from the Variance Inflation Factor (VIF) from the multivariable model for the San Pedro de Macoris data set, Dominican Republic, 2021.**

| **Variable** | **VIF** | **95%CI** |
| --- | --- | --- |
| Age | 1·54 | (1·44–1·67) |
| Gender | 1·34 | (1·26–1·45) |
| River density | 1·16 | (1·10–1·25) |
| Motorized travel time to health facilities | 5·57 | (5·06–6·15) |
| GDP | 1·76 | (1·64–1·91) |
| Crop area | 2·46 | (2·26–2·69) |
| Flooding risk | 1·37 | (1·29–1·47) |
| Rainfall | 5·19 | (4·71–5·73) |
| Ethnicity | 1·65 | (1·54–1·79) |
| Work environment | 1·66 | (1·55–1·8) |
| Exposure to freshwater | 1·44 | (1·35–1·56) |
| Educational level | 1·20 | (1·14–1·30) |
| Contact with rat | 1·83 | (1·70–1·99) |

###

### Final set of variables included in the province-specific models

**Supplementary Table 7. Spatial data sources and encoding methods for environmental and sociodemographic covariates included in the final multivariable models generated to investigate leptospirosis seropositivity in the Dominican Republic, Jun-Oct 2021.**

| Description and spatial resolution | Encoding | Sources |
| --- | --- | --- |
| Flooding-risk area | | |
| A vector layer with a national level map delimitating areas considered to be at risk of flooding. | Geolocations of households were overlayed with the flooding-risk map and classified as located at the flooding-risk area or not. | Provided by Gregorio Rosario Michel^1^ |
| River density | | |
| A polyline layer with national level map of main rivers. | Espaillat - a 250-metre buffer was created for each household. The total length of rivers (metres) was extracted.  SPM^2^ - a 500-metre buffer was created for each household. The total length of rivers (metres) was extracted. | WorldPop Project (4) |
| Motorized time travel to healthcare facilities | | |
| Raster file at the national level (~1km grid), with estimates of average travel time (in minutes) to the nearest hospital or clinic by motorized transport. | Geolocations of households were overlayed with the raster and the pixel value was extracted. | Malaria Atlas Project (7) |
| Gross Domestic Product (GDP) | | |
| Raster file at the national level (~1km grid) with the average GDP given in 1,000,000 USD (2011) per capita. | Geolocations of households were overlayed with the raster and the pixel value was extracted. For households with missing data, a 1km buffer was created, overlaying a 10x10 grid. GDP values were extracted for each point of the grid and the average value was given to the household with missing data. | Global Human Settlement (11) |
| Cropland area and Bare ground | | |
| A raster file (10 metres grid) of the global LULC^3^ map with 11 LULC classes was used to generate six separate rasters for the LULC categories that cover the DR: crops, rangelands, bare ground, trees, flooded vegetation and built/urban area. Each LULC raster files was transformed in a vector layer. | A 250-metre buffer was created for each household and overlayed with each LULC vector map. The percentage of each LULC occupying the household 250m buffer area was extracted. | Sentinel-2 Global Land Use/Land Cover (LULC) - Esri (13). |
| Rainfall | | |
| Raster files at the national level (~11km grid) with total precipitation (mm) per month. | Geolocations of households were overlayed with the raster and the pixel value was extracted. | CHIIRPS (10) |

^1^Maps created by National Geologic Service and provided by Dr Gregorio Antonio Rosario Michel, ^2^SPM - San Pedro de Macoris, ^3^LULC - Land Use/Land Cover.

## Generalised additive model and variable aggregation

Continuous variables selected to be included in the Province-specific models were further explored to identify nonlinear association using bivariate generalised additive models (GAM). For variables in which a nonlinear association was identified, the variable density distribution and quartile distribution were explored to identify the optimal aggregation to create a categorised variable.

Espaillat

- 1. Motorized travel time to health facilities (strong nonlinear association - estimated degrees of freedom (EDF) 5.5

**Figure 7. Estimated Smooth Effect of motorised time travel to health facilities on Log-Odds of Seropositivity from Generalised Additive Model, Espaillat, Jun-Oct 2021:
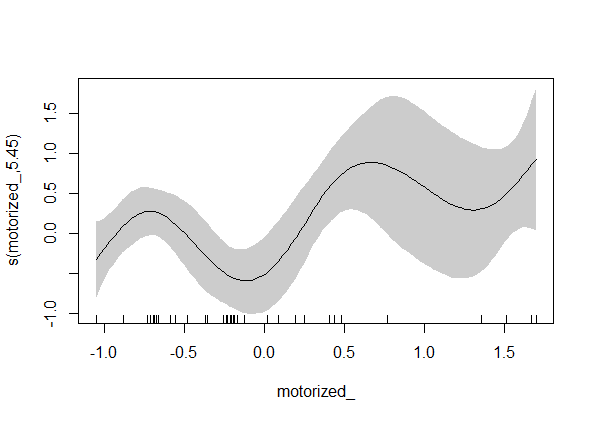
**

**Table 8. Motorized travel time to health facilities (in minutes) interquartile distribution, Espaillat, Jun-Oct 2021:**

| 1^st^ IQR | 2^nd^ IQR | 3^rd^ IQR | 4^th^ IQR |
| --- | --- | --- | --- |
| 0.00 – 1.37 | 1.38 – 2.85 | 2.86 – 4.68 | 4.69 – 11.32 |

- 1. GDP – linear association (EDF 1.0)

**Figure 8. Estimated Smooth Effect of GDP on Log-Odds of Seropositivity from Generalised Additive Model, Espaillat, Jun-Oct 2021:**


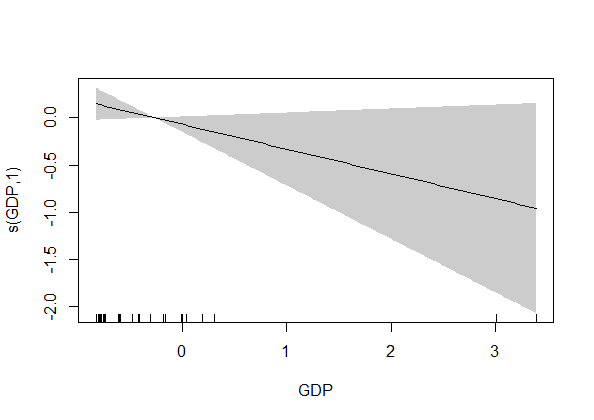


- 1. Bare ground – nonlinear association (EDF 4.2)

**Figure 9. Estimated Smooth Effect of bare ground coverage on Log-Odds of Seropositivity from Generalised Additive Model, Espaillat, Jun-Oct 2021:**


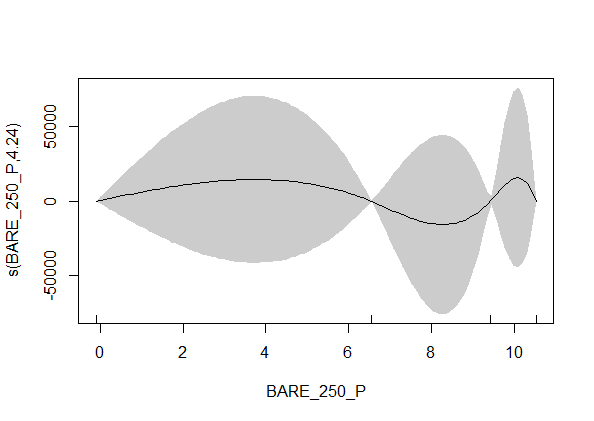


**Table 9. Percentage of area covered by bare ground in a 250m buffer surrounding each household interquartile distribution, Espaillat, Jun-Oct 2021:**

| 1^st^ IQR | 2^nd^ IQR | 3^rd^ IQR | 4^th^ IQR |
| --- | --- | --- | --- |
| 0.00 – 0.00 | 0.00 – 0.00 | 0.00 – 0.00 | 0.00 – 2.38 |

**Figure 10. Density distribution og bare ground distribution among participants, Espaillat, Jun-Oct 2021:**


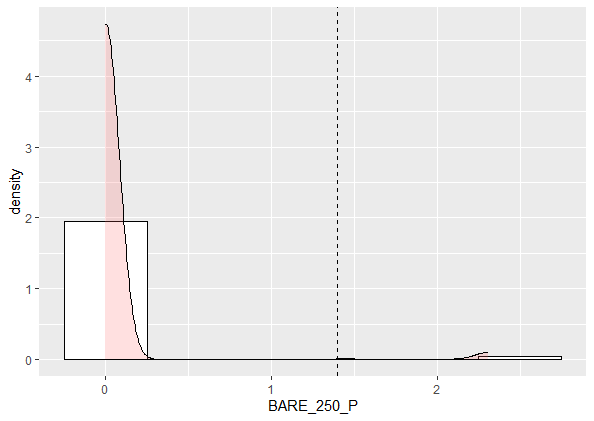


The dasline (1.44%) represents the break point used to aggregate data. A total of 782 participants had <1.4 % the 250m buffer area covered by bare ground, and 20 participants had >1.4% (mean 0.05%).

- 1. Crop area – strong nonlinear association (EDF 6.8)

**Figure 11. Estimated Smooth Effect of crop land coverage on Log-Odds of Seropositivity from Generalised Additive Model, Espaillat, Jun-Oct 2021:**


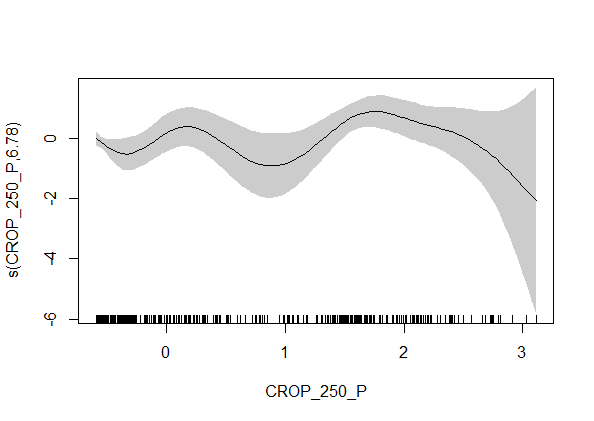


**Table 10. Percentage of area covered by crop land in a 250m buffer surrounding each household interquartile distribution, Espaillat, Jun-Oct 2021:**

| 1^st^ IQR | 2^nd^ IQR | 3^rd^ IQR | 4^th^ IQR |
| --- | --- | --- | --- |
| 0.00 – 0.00 | 0.00 – 3.85 | 3.86 – 42.15 | 42.16 – 87.85 |

- 1. Rainfall - strong nonlinear association (EDF 5.7)

**Figure 12. Estimated Smooth Effect of 5-year average precipitation on Log-Odds of Seropositivity from Generalised Additive Model, Espaillat, Jun-Oct 2021:**


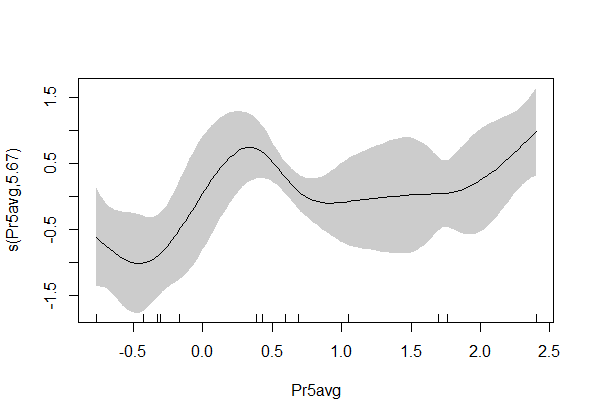


**Table 11. Average 5-year precipitation interquartile distribution, Espaillat, Jun-Oct 2021:**

| 1^st^ IQR | 2^nd^ IQR | 3^rd^ IQR | 4^th^ IQR |
| --- | --- | --- | --- |
| 80.00 - 85.50 | 85.51 - 86.59 | 86.59 88.68 | 88.68 95.14 |

- 1. River density - strong nonlinear association (EDF 5.5)

**Figure 13. Estimated Smooth Effect of river density (total length in a 250m buffer) on Log-Odds of Seropositivity from Generalised Additive Model, Espaillat, Jun-Oct 2021:**


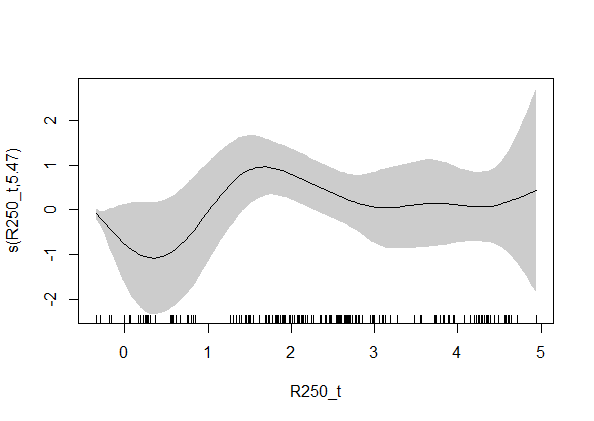


**Table 12. Total river length in a 250m buffer surrounding each household interquartile distribution, Espaillat, Jun-Oct 2021:**

| 1^st^ IQR | 2^nd^ IQR | 3^rd^ IQR | 4^th^ IQR |
| --- | --- | --- | --- |
| 0.00 – 0.00 | 0.00 - 0.00 | 0.00 235.6 | 235.7 1048.0 |

San Pedro de Macoris

- 1. River density – nonlinear association (EDF 4.6)

**Figure 14. Estimated Smooth Effect of river density (total length in a 500m buffer) on Log-Odds of Seropositivity from Generalised Additive Model, San Pedro de Macorís, Jun-Oct 2021:**


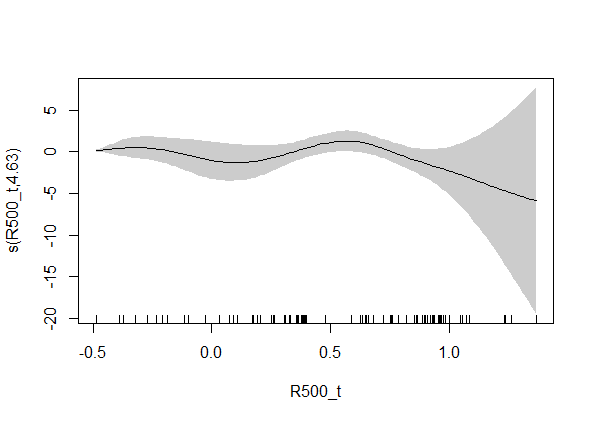


**Table 13. Total river length in a 500m buffer surrounding each household interquartile distribution, San Pedro de Macorís, Jun-Oct 2021:**

| 1^st^ IQR | 2^nd^ IQR | 3^rd^ IQR | 4^th^ IQR |
| --- | --- | --- | --- |
| 0.00 – 0.00 | 0.00 – 0.00 | 0.00 – 0.00 | 0.00 – 1059.70 |

**Figure 15. Density distribution of river desnity distribution among participants, San Pedro de Macorís, Jun-Oct 2021:**


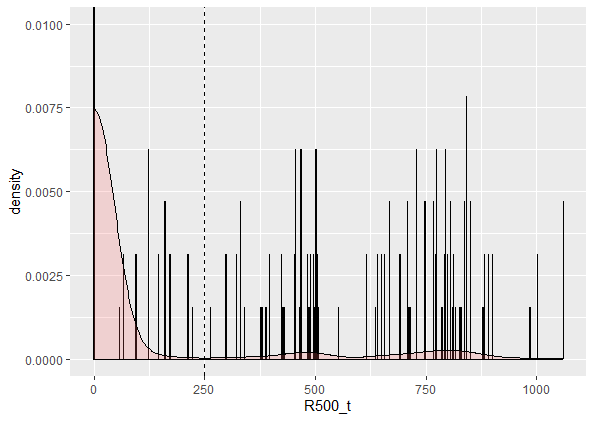


The dashline (250m) represents the break point at which the data was aggregated. A total of 1152 participants had <250m length of rivers in a 500m buffer, and 124 participants >250 m (mean 74.8 m).

- 1. Motorized travel time to health facilities – strong nonlinear association (5.5)

**Figure 16. Estimated Smooth Effect of motorized travel time to health facilities (in minutes) on Log-Odds of Seropositivity from Generalised Additive Model, San Pedro de Macorís, Jun-Oct 2021:**


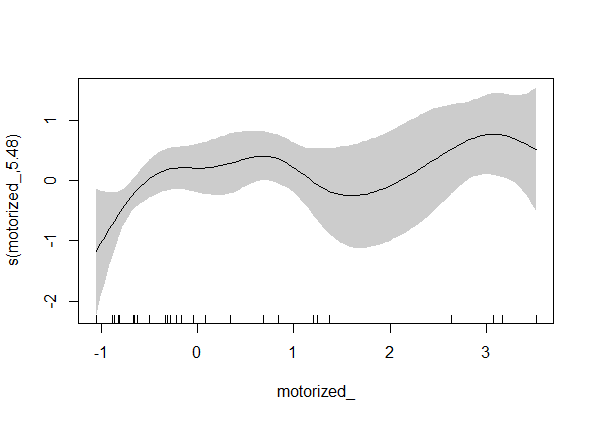


**Table 14. Motorized time travel to health facilities (in minutes) interquartile distribution, San Pedro de Macorís, Jun-Oct 2021:**

| 1^st^ IQR | 2^nd^ IQR | 3^rd^ IQR | 4^th^ IQR |
| --- | --- | --- | --- |
| 0.00 – 1.57 | 1.58 - 3.40 | 3.41 7.15 | 7.16 18.78 |

- 1. GDP – strong nonlinear association (EDF 5.9)

**Figure 17. Estimated Smooth Effect of GDP on Log-Odds of Seropositivity from Generalised Additive Model, San Pedro de Macorís, Jun-Oct 2021**


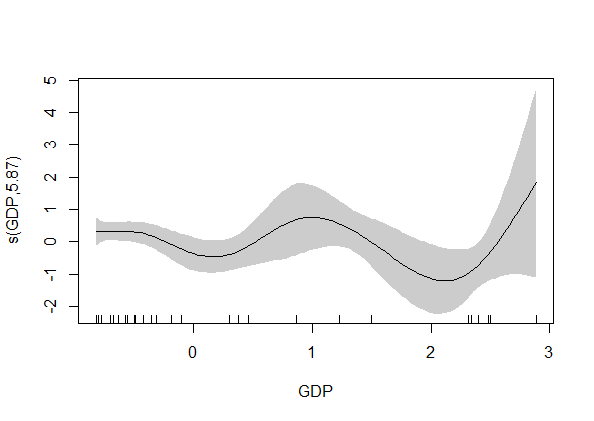


**Table 15. GDP interquartile distribution, San Pedro de Macorís, Jun-Oct 2021:**

| 1^st^ IQR | 2^nd^ IQR | 3^rd^ IQR | 4^th^ IQR |
| --- | --- | --- | --- |
| 0.03 – 2.08 | 2.09 - 5.64 | 6.65 14.20 | 14.21 40.96 |

- 1. Crop area – linear association (EDF 1.0)

**Figure 18. Estimated Smooth Effect of crop land coverage in a 250m buffer on Log-Odds of Seropositivity from Generalised Additive Model, San Pedro de Macorís, Jun-Oct 2021**


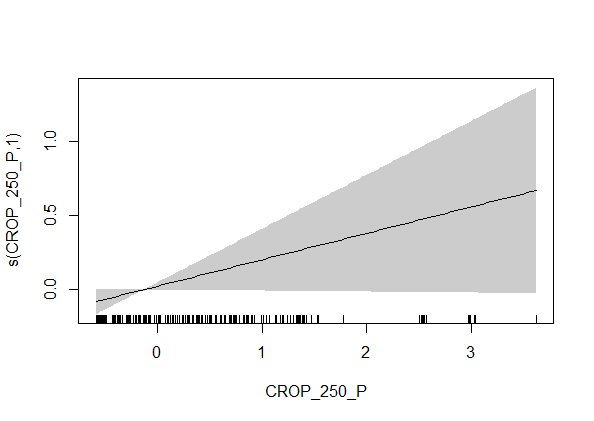


- 1. Rainfall – linear association (EDF 1.0)

**Figure 19. Average 5-year precipitation on Log-Odds of Seropositivity from Generalised Additive Model, San Pedro de Macorís, Jun-Oct 2021**


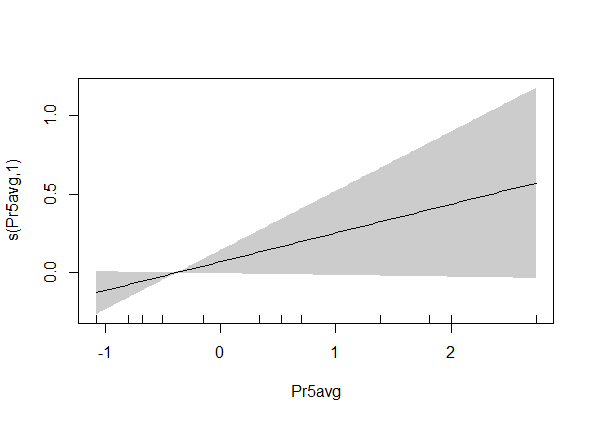


Mixed-effect model and intra-class correlation

The intra-class correlation was estimated using a latetnt variable approach, as the residual variance is not estimated for binary outcomes (Bernouli distribution). As the variance at the individual level is estimated to be:

$$\sigma^{2}=\frac{\pi^{2}}{3} \approx3.29$$

Then:

$$ICC=\frac{\sigma_{cluster}^{2}}{\sigma_{cluster}^{2}+\frac{\pi^{2}}{3}}$$

Results from the GLMER models with BHS, had an estimated ICC of 0.714 (CrI95% 0.493 - 0.838) for the Espaillat specific model, and 0.570 (CrI95% 0.273 - 0.759) for SPM.

## Models’ performance

**Supplementary Table 8. Area Under the Receiver Operating Characteristic Curve (AUC) by statistical method and by province.**

|  | **GLMER** | **GGWR** |
| --- | --- | --- |
| **Espaillat** | 0.7294 | 0.7406707 |
| **San Pedro de Macoris** | 0.7673 | 0.7406707 |

# References:

1. Nilles EJ, Paulino CT, de St. Aubin M, Restrepo AC, Mayfield H, Dumas D, et al. SARS-CoV-2 seroprevalence, cumulative infections, and immunity to symptomatic infection – A multistage national household survey and modelling study, Dominican Republic, June–October 2021. The Lancet Regional Health - Americas. 2022;16:100390.

2. Nilles E, de St. Aubin M, Dumas D, Duke W, Etienne MC, Abdalla G, et al. Monitoring Temporal Changes in SARS-CoV-2 Spike Antibody Levels and Variant-Specific Risk for Infection, Dominican Republic, March 2021–August 2022. Emerging Infectious Disease journal. 2023;29(4):723.

3. Oficina Nacional de Estadistica. Oficina Nacional de Estadisticas - Datos y Estadisticas [Available from: <https://www.one.gob.do/>.

4. WorldPop. Global High Resolution Population Denominators Project: Funded by The Bill and Melinda Gates Foundation (OPP1134076). ; 2018 [updated 06/04/2019. Available from: <https://hub.worldpop.org/>.

5. (ESRI). ESRI. ArcGIS Software version 10.7.1.: Redlands, California; 2019.

6. OpenStreetMap. Distance to Education Facilities: OpenStreetMap Foundation [Available from: <https://www.openstreetmap.org/>.

7. Malaria Atlas. Travel Time to nearest healthcare facility with access to motorized transport. 2019 [Available from: <https://malariaatlas.org/>.

8. Farr TG, Kobrick, M., . Shuttle Radar Topography Mission produces a wealth of data. Eos Trans. AGU,2000. p. 583-.

9. (NASA). TNAaSA. Land Surface Temperature and Emissivity (LT&E) (MOD11A2). 2016.

10. Funk C, Peterson P, Landsfeld M, Pedreros D, Verdin J, Shukla S, et al. The climate hazards infrared precipitation with stations—a new environmental record for monitoring extremes. Scientific Data. 2015;2(1):150066.

11. Kummu M, Taka M, Guillaume JHA. Gridded global datasets for Gross Domestic Product and Human Development Index over 1990-2015. Sci Data. 2018;5:180004.

12. Kummu M, Taka M, Guillaume JHA. Data from: Gridded global datasets for Gross Domestic Product and Human Development Index over 1990-2015 [Dataset]. In: Dryad., editor. 2020.

13. Karra K, et al. Global land use/land cover with Sentinel-2 and deep learning. In: IGARSS 2021-2021, editor. IEEE International Geoscience and Remote Sensing Symposium.: IEEE, 2021; 2022.

14. Rosario Michel G, Muñoz Tapia S, Manzano Aybar F, Guzmán Javier V, Crompvoets J. Identifying Users’ Requirements for Emergency Mapping Team Operations in the Dominican Republic. ISPRS International Journal of Geo-Information. 2020;9(3):165.

15. United Nations Office for the Coordination of Humanitarian Affairs (OCHA). Dominican Republic - Subnational Administrative Boundaries (COD-AB) [Data set]: Humanitarian Data Exchange; 2024 [Available from: <https://data.humdata.org/dataset/cod-ab-dom>.
